# Enolase represents a metabolic checkpoint controlling the differential exhaustion programmes of hepatitis virus-specific CD8^+^ T cells

**DOI:** 10.1136/gutjnl-2022-328734

**Published:** 2023-08-04

**Authors:** Frances Winkler, Anna V Hipp, Carlos Ramirez, Bianca Martin, Matteo Villa, Emilia Neuwirt, Oliver Gorka, Jeroen Aerssens, Susanne E Johansson, Nisha Rana, Sian Llewellyn-Lacey, David A Price, Marcus Panning, Olaf Groß, Erika L Pearce, Carl M Hermann, Kathrin Schumann, Luciana Hannibal, Christoph Neumann-Haefelin, Tobias Boettler, Percy Knolle, Maike Hofmann, Dirk Wohlleber, Robert Thimme, Bertram Bengsch

**Affiliations:** 1 Clinic for Internal Medicine II, Freiburg University Medical Center, Faculty of Medicine, University of Freiburg, Freiburg im Breisgau, Germany; 2 Faculty of Biology, University of Freiburg, Freiburg im Breisgau, Germany; 3 Health Data Science Unit, Medical Faculty, University of Heidelberg, Heidelberg, Germany; 4 Max Planck Institute of Immunobiology and Epigenetics, Freiburg im Breisgau, Germany; 5 Institute of Neuropathology, Freiburg University Medical Center, Faculty of Medicine, University of Freiburg, Freiburg im Breisgau, Germany; 6 Signalling Research Centres BIOSS and CIBSS, University of Freiburg, Freiburg im Breisgau, Germany; 7 Translational Biomarkers, Infectious Diseases Therapeuic Area, Janssen Pharmaceutica, Beerse, Belgium; 8 Division of Infection and Immunity, Cardiff University School of Medicine, Cardiff University, Cardiff, UK; 9 Systems Immunity Research Institute, Cardiff University School of Medicine, Cardiff, UK; 10 Institute of Virology, Freiburg University Medical Center, Faculty of Medicine, University Hospital Freiburg, Freiburg im Breisgau, Germany; 11 The Bloomberg-Kimmel Institute for Cancer Immunotherapy, Johns Hopkins Medicine Sidney Kimmel Comprehensive Cancer Center, Baltimore, Maryland, USA; 12 Institute for Medical Microbiology, Immunology and Hygiene, Technical University of Munich (TUM), Munich, Germany; 13 Department of General Pediatrics, Laboratory of Clinical Biochemistry and Metabolism, Medical Center-University of Freiburg, Adolescent Medicine and Neonatology, Faculty of Medicine, University of Freiburg, Freiburg, Germany; 14 German Center for Infection Research (DZIF), Munich Partner Site, Munich, Germany; 15 Institute of Molecular Immunology, Klinikum Rechts der Isar, Technical University of Munich, Munich, Germany; 16 German Cancer Consortium (DKTK), Partner Site Freiburg, Heidelberg, Germany

**Keywords:** chronic viral hepatitis, hepatitis B, hepatitis C, alpha beta T cells, immunology in hepatology

## Abstract

**Objective:**

Exhausted T cells with limited effector function are enriched in chronic hepatitis B and C virus (HBV and HCV) infection. Metabolic regulation contributes to exhaustion, but it remains unclear how metabolism relates to different exhaustion states, is impacted by antiviral therapy, and if metabolic checkpoints regulate dysfunction.

**Design:**

Metabolic state, exhaustion and transcriptome of virus-specific CD8^+^ T cells from chronic HBV-infected (n=31) and HCV-infected patients (n=52*)* were determined *ex vivo* and during direct-acting antiviral (DAA) therapy. Metabolic flux and metabolic checkpoints were tested *in vitro*. Intrahepatic virus-specific CD8^+^ T cells were analysed by scRNA-Seq in a HBV-replicating murine *in vivo* model of acute and chronic infection.

**Results:**

HBV-specific (core_18-27_, polymerase_455-463_) and HCV-specific (NS3_1073-1081_, NS3_1406-1415_, NS5B_2594-2602_) CD8^+^ T cell responses exhibit heterogeneous metabolic profiles connected to their exhaustion states. The metabolic state was connected to the exhaustion profile rather than the aetiology of infection. Mitochondrial impairment despite intact glucose uptake was prominent in severely exhausted T cells linked to elevated liver inflammation in chronic HCV infection and in HBV polymerase_455-463_ -specific CD8^+^ T cell responses. In contrast, relative metabolic fitness was observed in HBeAg-negative HBV infection in HBV core_18-27_-specific responses. DAA therapy partially improved mitochondrial programmes in severely exhausted HCV-specific T cells and enriched metabolically fit precursors. We identified enolase as a metabolic checkpoint in exhausted T cells. Metabolic bypassing improved glycolysis and T cell effector function. Similarly, enolase deficiency was observed in intrahepatic HBV-specific CD8^+^ T cells in a murine model of chronic infection.

**Conclusion:**

Metabolism of HBV-specific and HCV-specific T cells is strongly connected to their exhaustion severity. Our results highlight enolase as metabolic regulator of severely exhausted T cells. They connect differential bioenergetic fitness with distinct exhaustion subtypes and varying liver disease, with implications for therapeutic strategies.

**WHAT IS ALREADY KNOWN ON THIS TOPIC**

- Accumulation of exhausted CD8^+^ T cells in patients with chronic hepatitis B and C virus (HBV and HCV) infection contributes to the failure to clear viral infection.
- These exhausted T cells lack sufficient antiviral function but the mechanisms behind this dysfunction are unclear.

**WHAT THIS STUDY ADDS**

- HBV-specific and HCV-specific CD8^+^ T cells exhibit distinct metabolic profiles that correlate with severity of exhaustion.
- Severe mitochondrial depolarisation despite high glucose uptake is present in severely exhausted virus-specific CD8^+^ T cells.
- HBV polymerase_455-463_-specific CD8^+^ T cells in cHBV infection display more severe exhaustion and mitochondrial dysfunction that correlates with quantitative HB surface antigen levels in contrast to HBV core_18-27_-specific CD8^+^ T cells.
- Enolase is a metabolic checkpoint limiting glycolytic flux in HBV-specific and HCV-specific CD8^+^ T cells.
- Effector function of enolase-inhibited CD8^+^ T cells is boosted by pyruvate supplementation.

**HOW THIS STUDY MIGHT AFFECT RESEARCH, PRACTICE OR POLICY**

- We identified the glycolytic enzyme enolase as a metabolic checkpoint that can restrict mitochondrial metabolism and effector function of HBV-specific and HCV-specific CD8^+^ T cells.
- This knowledge points to interventions to enhance or bypass enolase activity in order to boost antiviral responses in chronic infection.
- Our data suggest that the exhaustion and metabolic state of HBV polymerase_455-463_-specific CD8^+^ T cells may act as correlates of differential antigen recognition in cHBV infection which should be further investigated.

## Introduction

Exhausted virus-specific CD8^+^ T cells (T_EX_) with limited effector function accumulate in patients with chronic hepatitis B and C virus infection (cHBV/cHCV).^1–4^ These exhausted T cells are characterised by increased PD-1 expression and other coinhibitory receptors and profound alterations in transcriptional programmes.^2 5 6^ Recent reports highlighted substantial heterogeneity among exhausted HBV-specific and HCV-specific CD8^+^ T cells, including early differentiated PD-1^+^CD127^+^ precursors of exhausted T cells with partial memory-like characteristics and more severely exhausted PD-1^hi^CD127^-^ T cells.^5 7–10^ Interestingly, many cellular alterations of exhausted virus-specific CD8^+^ T cells persist even after antiviral therapy.^7 10–13^ However, the precise mechanisms determining these exhausted T cell programmes remain poorly defined.

T cell energy metabolism and effector T cell function are tightly interconnected. Specific roles of glycolytic enzymes in T cell activation and cytokine production have been described, such as glyceraldehyde phosphate dehydrogenase (GAPDH),^14 15^ pyruvate dehydrogenase kinase 1 (PDHK1)^16^ or the glycolytic metabolite phosphoenolpyruvate (PEP).^17^ Conversely, regulation of the metabolic flux may explain limited T cell function. Indeed, bioenergetic regulation downstream of inhibitory receptor signalling was identified as a major hallmark of exhausted T cells in the murine lymphocytic choriomeningitis virus (LCMV) model of chronic viral infection.^18^ This bioenergetic regulation affected glycolysis and resulted in a significant disorganisation of mitochondrial organelles with alterations in ultrastructure, membrane depolarisation and production of reactive oxygen species (ROS).^17–19^ Investigations of the metabolic properties of HBV-specific and HCV-specific CD8^+^ T cells identified related metabolic dysregulation. HBV-specific CD8^+^ T cells analysed *in vitro* were highly dependent on glycolysis, and unable to switch efficiently to oxidative phosphorylation (OXPHOS) in settings of glucose restriction.^20^ Fisicaro *et al.* identified that significant downregulation of mitochondrial function is connected to ROS production in exhausted HBV-specific CD8^+^ T cells - comparable to the findings in LCMV infection.^21^ HBV-specific CD8^+^ T cells targeting different epitopes, such as HBV core_18-27_-specific or polymerase_455-463_-specific responses differ in their exhaustion phenotype^9^ but their metabolic properties are unknown. Mitochondrial function was also investigated in HCV-specific CD8^+^ T cells and during direct-acting antiviral (DAA) therapy. While one study reported limited changes in mitochondrial function of HCV-specific CD8^+^ T cells after DAA therapy and HCV clearance,^11^ another study observed a reduction of cells with depolarised mitochondria.^22^ However, currently, it remains unclear whether there are differences in the metabolic regulation of HBV-specific and HCV-specific CD8^+^ T cells, how these relate to the severity of exhaustion programmes, whether they change during antiviral therapy, and if specific glycolytic checkpoints are involved.

To address these important questions, we performed a detailed analysis of the metabolic profiles of HBV-specific and HCV-specific CD8^+^ T cells and their exhaustion states. We observed major differences in the exhaustion and metabolic programmes of HBV-specific and HCV-specific CD8^+^ T cells. HCV-specific responses were enriched for more severe exhaustion phenotypes and connected to more pronounced mitochondrial impairment despite high glucose uptake. However, a similar mitochondrial impairment was observed in HBV polymerase_455-463_-specific CD8^+^ T cells but not in HBV core_18-27_-specific CD8^+^ T cells during HBeAg-negative infection. These differences were connected to more severe exhaustion and in cHCV to a higher level of liver inflammation. In HBV infection, a correlation between HBV polymerase_455-463_-specific metabolism and quantitative HB surface antigen (qHBsAg) levels was observed. Antigen removal *in vivo* during DAA therapy partially improved the metabolism of the more severely exhausted T cell subset. Enolase was identified as a metabolic checkpoint. Its reduced expression in severely exhausted CD8^+^ T cells is involved in regulation of the glycolytic flux, contributing to metabolic dysfunction and reduced antiviral effector function. Bypassing this metabolic bottleneck reinvigorated effector function of virus-specific CD8^+^ T cells. Enolase deficiency was a conserved feature of severely exhausted murine intrahepatic HBV-specific CD8^+^ T cells in chronic but not acute infection. Together, these data highlight differential metabolic programming of virus-specific CD8^+^ T cells in different exhausted T cell subsets in viral hepatitis and highlight a novel metabolic checkpoint.

## Methods

### Study cohort

Patient details are summarised in online supplemental tables 1 and 2.

10.1136/gutjnl-2022-328734.supp1 Supplementary data

### Peptides, tetramers and antibodies

HLA-A*02:01-restricted monomers of immunodominant epitopes for HBV (HLA-A*02:01/core_18-27_: FLPSDFFPSV; HLA-A*02:01/polymerase_455-463_: GLSRYVARL), HCV (HLA-A*02:01/NS3_1073-1081_: CINGVCWTV; HLA-A*02:01/NS3_1406-1415_: KLVALGINAV; HLA-A*02:01/NS5B_2594-2602_: ALYDVVSKL), EBV (HLA-A*02:01/BMLF1_280-288_: GLCTLVAML), CMV (HLA-A*02:01/pp65_495-503_: NLVPMVATV) and FLU (HLA-A*02:01/M1_58-66_: GILGFVFTL) were synthesised and conjugated with allophycocyanin (APC)-labelled or phycoerythin (PE)-labelled Streptavidin (Agilent) in a molar 4:1 ratio. Antibodies used are listed in online supplemental table 3.

### Statistics

**Figure 1:**
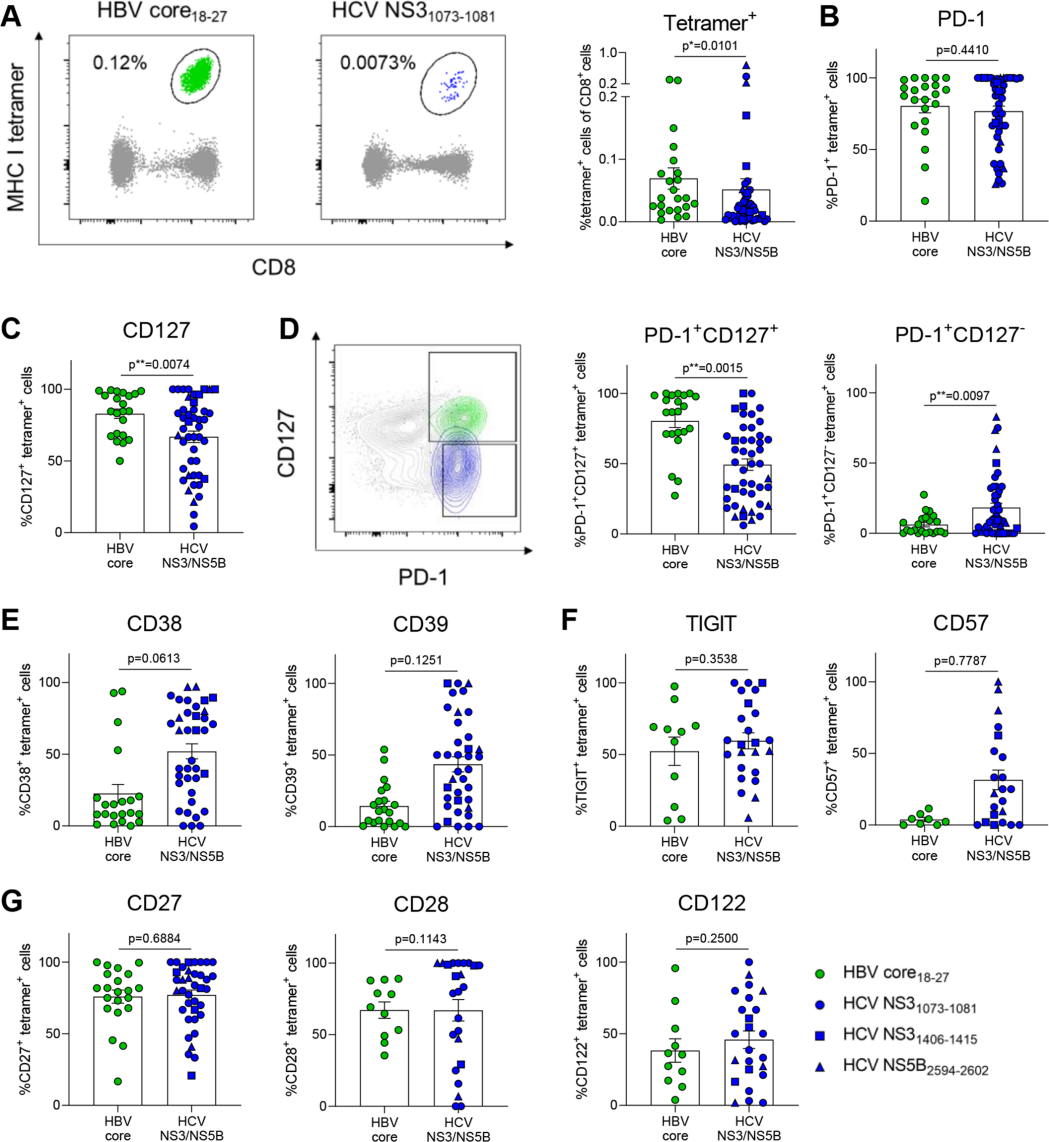
HCV-specific CD8^+^ T cells express markers associated with severe exhaustion. (A) Tetramer analysis of PBMCs from therapy-naïve cHBV and cHCV patients. Representative FACS plots of HBV core_18-27_- and HCV NS3_1073-1081_-specific CD8^+^ T cells (left). Comparison of tetramer frequencies gated on CD8^+^ T cells (right). Virus-specific CD8^+^ T cells from cHBV and cHCV patients were stained for the depicted exhaustion-associated molecules. (B, C) Frequencies of PD-1^+^, CD127^+^, (D) PD-1^+^CD127^+^, PD-1^+^CD127^-^, (E) CD38^+^, CD39^+^, (F) TIGIT^+^, CD57^+^, (G) CD27^+^, CD28^+^ and CD122^+^ virus-specific CD8^+^ T cells are indicated. HBV core_18-27_ (green) and HCV NS3_1073-1081_ epitopes (blue) are represented by circles. HCV NS3_1406-1415_ and HCV NS5B_2594-2602_ epitopes are visualised by blue squares and triangles, respectively. Mann-Whitney test was performed in (A, B), (D, E) and (G, left). Unpaired t-test was performed in (C), (F, G, centre and right). P values are indicated (p*<0.05, p**<0.01). Error bars indicate mean±SEM. cHBV, chronic hepatitis B virus; cHCV, chronic hepatitis C virus; PBMCs, peripheral blood mononuclear cells.

**Figure 2:**
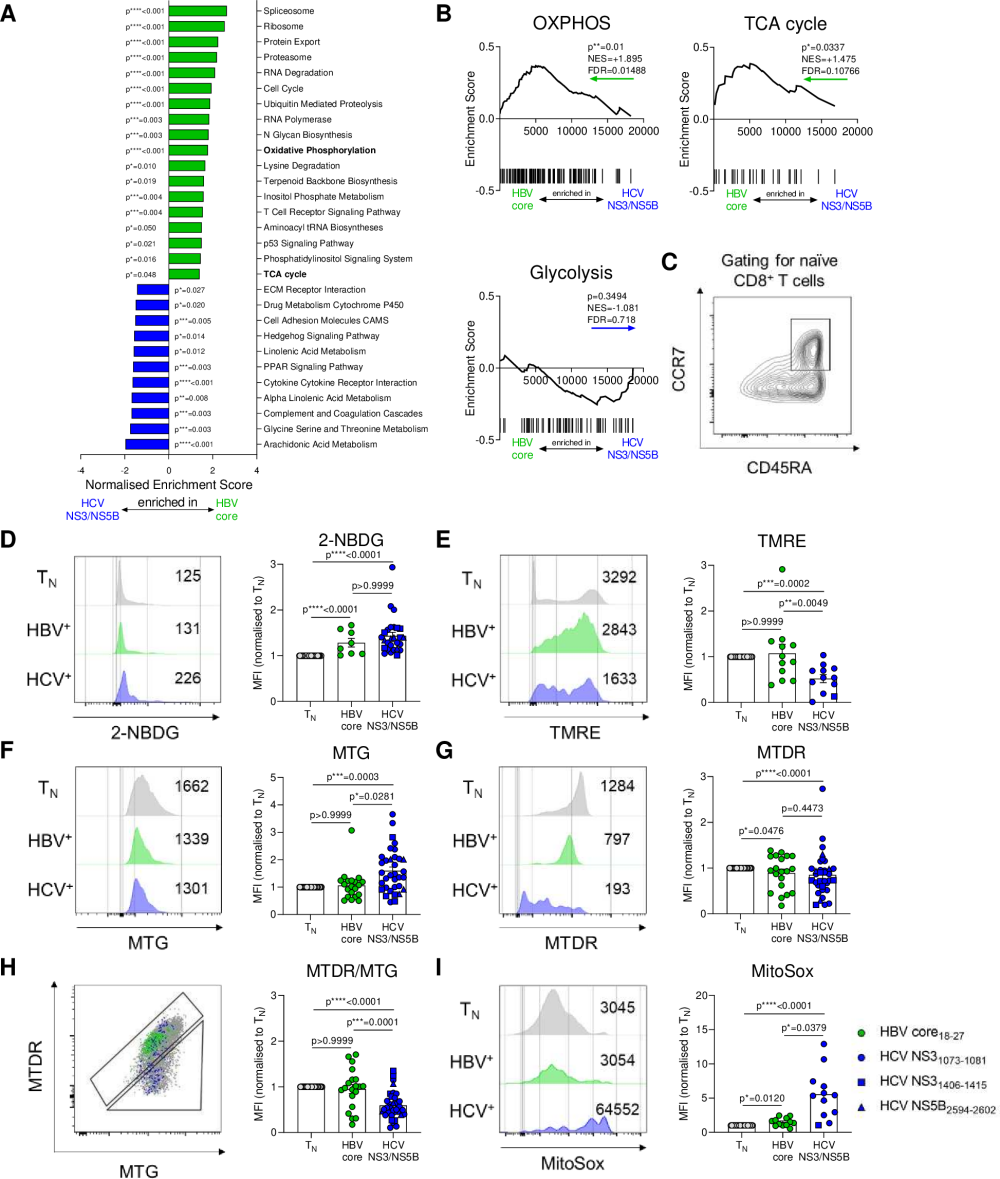
HBV-specific and HCV-specific CD8^+^ T cells display different metabolic profiles. (A) Gene-set enrichment analyses (GSEA) of KEGG metabolic pathways were performed on microarray data of sorted therapy-naïve HBV-specific and HCV-specific CD8^+^ T cells. Significant (p≤0.05) pathways were indicated. (B) GSEA plots for KEGG OXPHOS, TCA cycle and Glycolysis gene sets. Coloured arrows indicate enrichment in HBV-specific or HCV-specific CD8^+^ T cells. Normalised enrichment scores (NES), false discovery rates (FDR) and p values are shown for GSEA analyses. (C) Naïve CD8^+^ T cells used as normalisation control for metabolic stainings were gated as CCR7^+^CD45RA^+^. (D) Virus-specific CD8^+^ T cells were analysed for metabolic features by staining for glucose uptake (2-NBDG), (E) mitochondrial membrane potential (TMRE), (F) mitochondrial mass (MTG), (G) mitochondrial mass and potential (MTDR), (H) polarisation (MTDR/MTG) and (I) mitochondrial ROS (MitoSox). Metabolic staining intensity was normalised to naïve CD8^+^ T cells from the same sample. Exemplary histograms of metabolic stainings are shown. HBV core_18-27_ (green) and HCV NS3_1073-1081_ epitopes (blue) are represented by circles. HCV NS3_1406-1415_ and HCV NS5B_2594-2602_ epitopes are visualised by blue squares and triangles, respectively. Kruskal-Wallis test was performed in (D–I). P values are indicated (p*<0.05, p**<0.01, p***<0.005, p****<0.001). Error bars indicate mean±SEM. HBV, hepatitis B virus; HCV, hepatitis C virus; ROS, reactive oxygen species.

**Figure 3:**
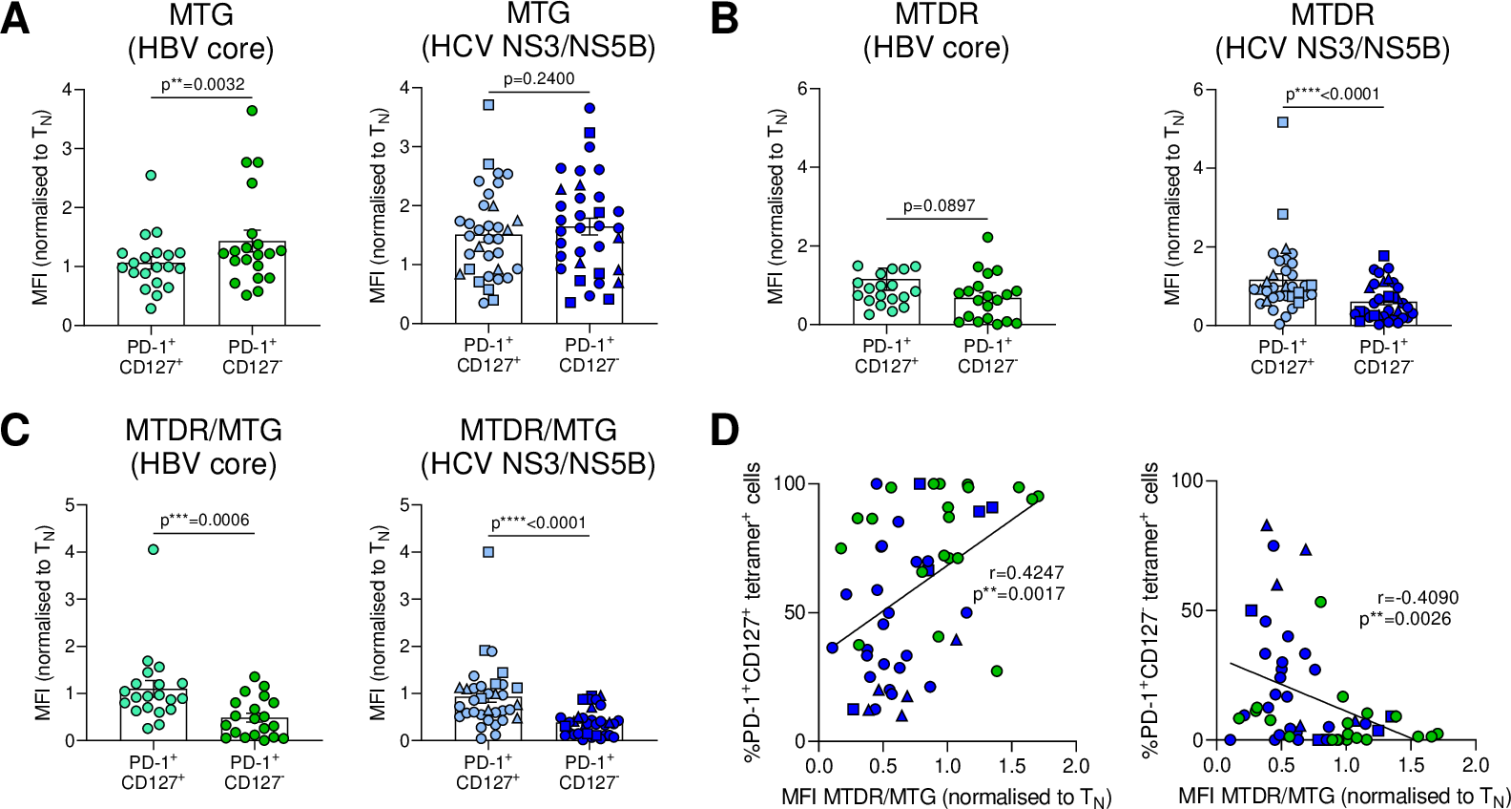
Severely exhausted virus-specific CD8^+^ T cells marked by PD-1^+^CD127^-^ phenotype show reduced mitochondrial polarisation. (A) PD-1^+^CD127^+^ and PD-1^+^CD127^-^ subsets of HBV-specific and HCV-specific CD8^+^ T cells were analysed *ex vivo* for mitochondrial mass (MTG), (B) mitochondrial mass and membrane potential (MTDR) and (C) mitochondrial polarisation (MTDR/MTG). (D) Correlation analyses of PD-1^+^CD127^+^ or PD-1^+^CD127^-^ virus-specific CD8^+^ T cells and their mitochondrial polarisation are shown. Metabolic staining intensity was normalised to naïve CD8^+^ T cells from the same sample. HBV core_18-27_ (green) and HCV NS3_1073-1081_ epitopes (blue) are represented by circles. HCV NS3_1406-1415_ and HCV NS5B_2594-2602_ epitopes are visualised by blue squares and triangles, respectively. Wilcoxon test was performed in (A, left) and (B, C). Paired t-test was performed in (A, right). Spearman r correlation analysis was performed in (D). P values are indicated (p**<0.01, p***<0.005, p****<0.001). Error bars indicate mean±SEM. HBV, hepatitis B virus; HCV, hepatitis C virus.

**Figure 4:**
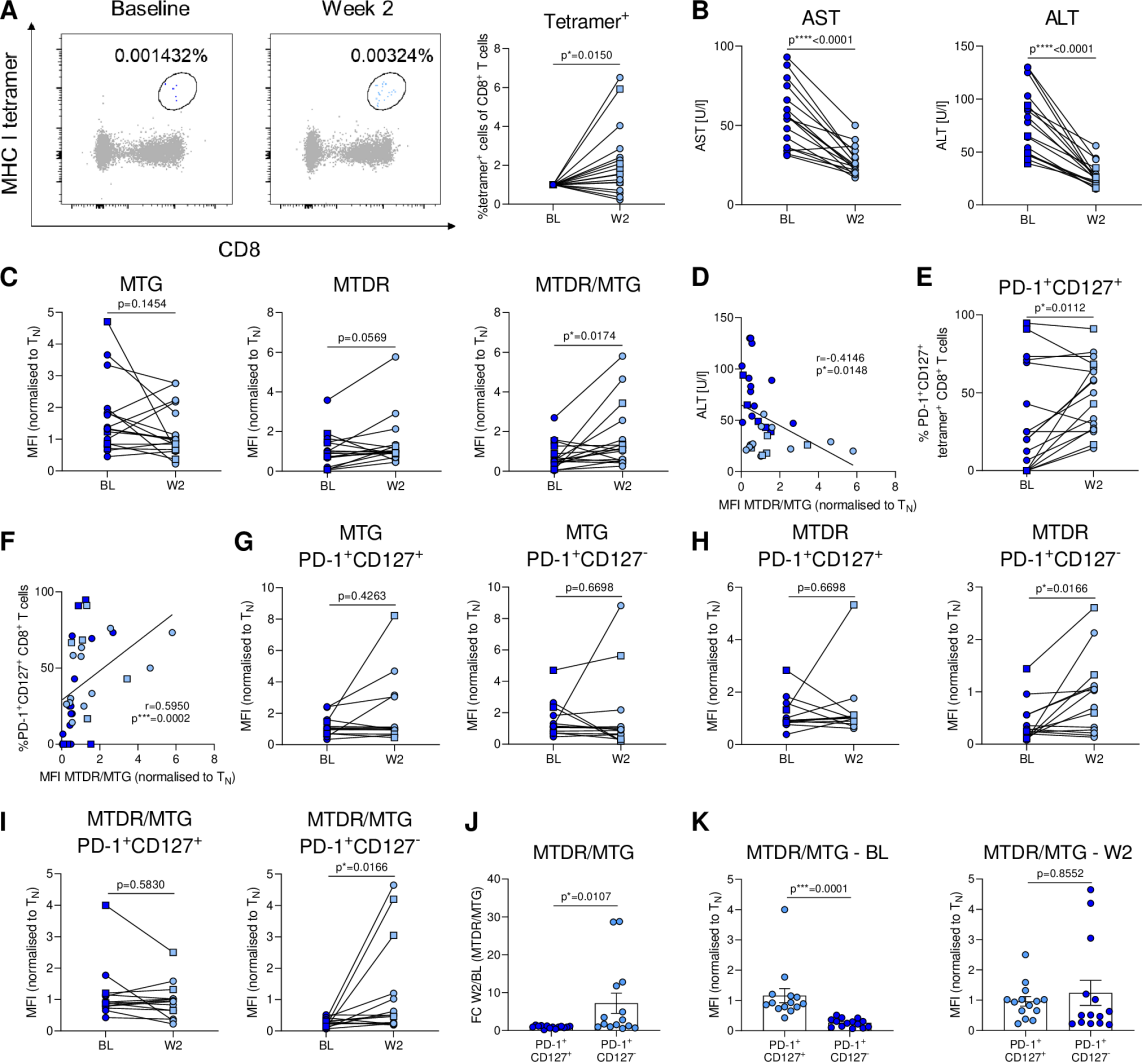
DAA therapy improves mitochondrial fitness of HCV-specific CD8^+^ T cells. cHCV patients were analysed before and during DAA therapy. (A) Tetramer analysis of HCV-specific CD8^+^ T cells at baseline (BL) and after 2 weeks of therapy (W2). (B) Serum transaminase levels at baseline and week 2. (C) Metabolic *ex vivo* staining of HCV-specific CD8^+^ T cells for mitochondrial mass (MTG), mitochondrial membrane potential (MTDR) and mitochondrial polarisation (MTDR/MTG) at BL and W2 time points. (D) Correlation analysis of ALT concentration and mitochondrial polarisation at BL and W2. (E) Frequency of PD-1^+^CD127^+^ HCV-specific CD8^+^ T cells at BL and W2. (F) Correlation of PD-1^+^CD127^+^ HCV-specific CD8^+^ T cells with mitochondrial function. *Ex vivo* FACS analysis of (G) MTG, (H) MTDR and (I) mitochondrial polarisation (MTDR/MTG) of PD-1^+^CD127^+^ or PD-1^+^CD127^-^ HCV-specific CD8^+^ T cells during DAA therapy. (J) Fold change of mitochondrial polarisation between W2 and BL was compared between PD-1^+^CD127^+^ and PD-1^+^CD127^-^ subsets. (K) Mitochondrial polarisation of PD-1^+^CD127^+^ and PD-1^+^CD127^-^ subsets at BL and W2 was compared. Metabolic staining intensity was normalised to naïve CD8^+^ T cells from the same sample. HCV NS3_1073-1081_ epitopes and HCV NS3_1406-1415_ epitopes are represented by blue circles and squares, respectively. Wilcoxon test was performed in (A–C) and (G–K). Paired t-test was performed in (E). Spearman r correlation analyses were performed in (D, F). P values are indicated (p*<0.05, p***<0.005, p****<0.001). Error bars indicate mean±SEM. DAA, direct-acting antiviral; HCV, hepatitis C virus.

**Figure 5:**
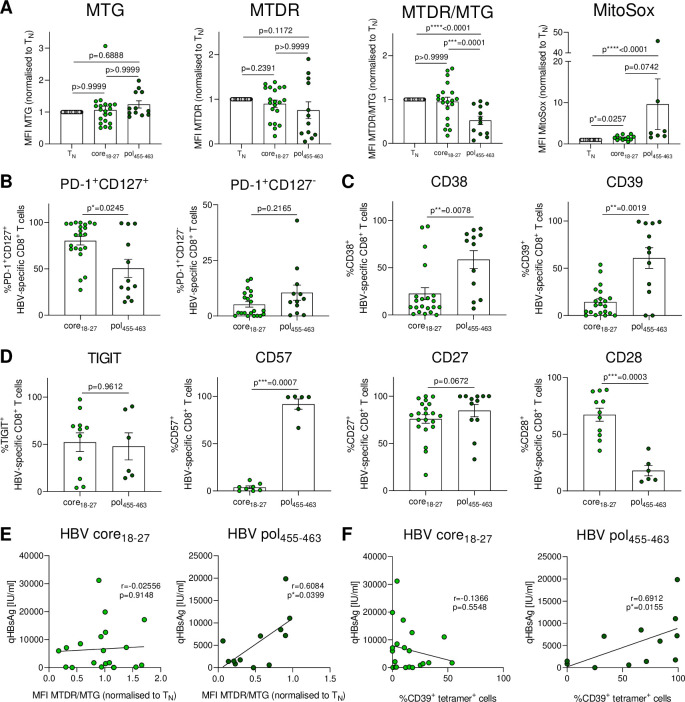
HBV core_18-27_- and polymerase_455-463_-specific CD8^+^ T cells exhibit distinct metabolic and exhaustion features and are differentially associated with viral load. (A) HBV-specific CD8^+^ T cells isolated from therapy-naïve cHBV patients were analysed *ex vivo* for mitochondrial mass (MTG), mitochondrial mass and membrane potential (MTDR), mitochondrial polarisation (MTDR/MTG), mitochondrial ROS (MitoSox) and (B) frequencies of PD-1^+^CD127^+^, PD-1^+^CD127^-^, (C) CD38^+^, CD39^+^, (D) TIGIT^+^, CD57^+^, CD27^+^ and CD28^+^ cells. (E) Correlation analyses of quantitative HBs antigen (qHBsAg) and mitochondrial polarisation and (F) frequencies of CD39^+^ tetramer^+^ cells are shown for HBV core_18-27_- and polymerase_455-463_-specific CD8^+^ T cells. Metabolic staining intensity was normalised to naïve CD8^+^ T cells from the same sample. HBV core_18-27_- and polymerase_455-463_-specific CD8^+^ T cells are visualised by light green and dark green circles, respectively. Kruskal-Wallis test was performed in (A). Mann-Whitney U test was performed in (B–D). Spearman r correlation analyses were perfomed in (E, F). P values are indicated (p*<0.05, p**<0.01, p***<0.005, p****<0.001). Error bars indicate mean±SEM. HBV, hepatitis B virus.

**Figure 6:**
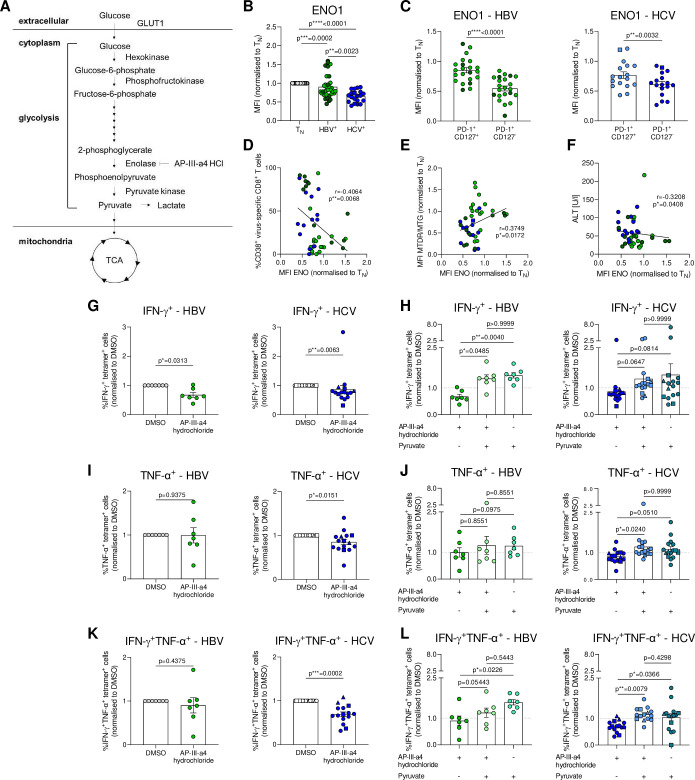
ENO1 lowly expressed in severely exhausted virus-specific CD8^+^ T cells is a metabolic checkpoint controlling glycolytic flux and T cell function. (A) Schematic overview of the glycolytic pathway. (B) Intracellular ENO1 staining of HBV-specific and HCV-specific CD8^+^ T cells. ENO1 MFIs are normalised to naïve CD8^+^ T cells of the respective donor. (C) *Ex vivo* analysis of PD-1^+^CD127^+^ and PD-1^+^CD127^-^ subsets of HBV-specific and HCV-specific CD8^+^ T cells for ENO1 expression. (D) Correlation analyses of ENO1 expression and frequencies of CD38^+^ virus-specific CD8^+^ T cells. (E) Correlation analyses of ENO1 expression and mitochondrial polarisation (MTDR/MTG) of HBV-specific and HCV-specific CD8^+^ T cells. (F) Correlation analysis of ENO1 expression and serum ALT level of therapy-naïve cHBV and cHCV patients. (G–L) IFN-γ and TNF-ɑ production of PMA-stimulated and ionomycin-stimulated hepatitis virus-specific CD8^+^ T cells after o/n treatment with DMSO, AP-III-a4 hydrochloride (10 µM) and/or sodium pyruvate (2 mM). Cytokine secretion is shown normalised to DMSO-treated hepatitis virus-specific CD8^+^ T cells as control samples. HBV core_18-27_- and polymerase_455-463_-specific CD8^+^ T cells are visualised by light green and dark green circles, respectively. HCV NS3_1073-1081_, HCV NS3_1406-1415_ and HCV NS5B_2594-2602_ epitopes are represented by blue circles, squares and triangles, respectively. Kruskall-Wallis test was performed in (B). Paired t-test was performed in (C). Spearman r correlation analyses were performed in (D–F). Wilcoxon test was performed in (G), (I, K). Friedman test was performed in (H), (J) and (L, left). Two-way ANOVA was performed in (L, right). P values are indicated (p*<0.05, p**<0.01, p***<0.005, p****<0.001). Error bars indicate mean±SEM. ANOVA, analysis of variance; cHBV, chronic hepatitis B virus; HCV, hepatitis C virus.

**Figure 7:**
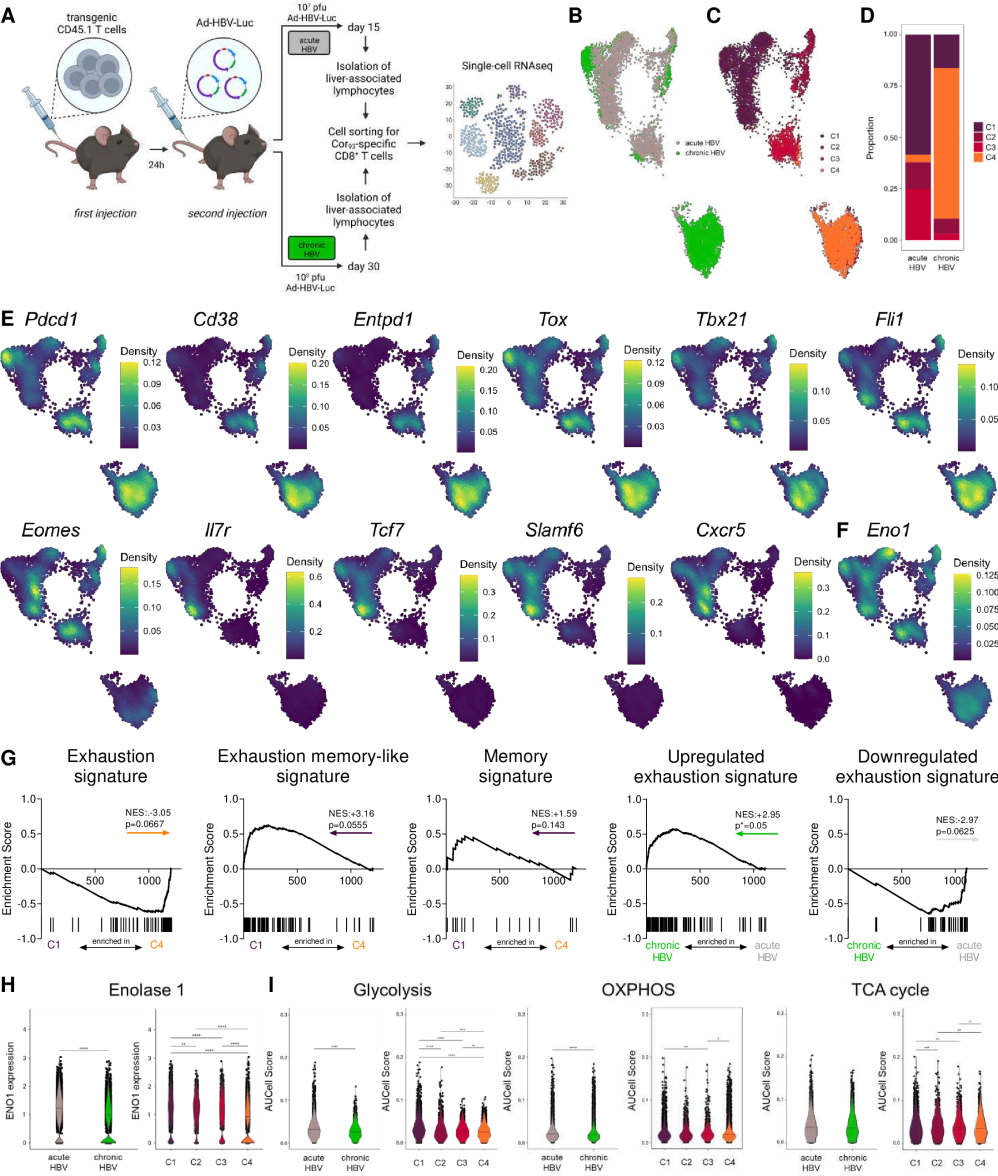
Differential enolase 1 expression is linked to different metabolic programmes and exhaustion severities in acute versus chronic HBV infection. (A) Schematic illustration depicting experimental procedure. C57Bl/6 mice were intravenously injected with 10 000 transgenic CD45.1 T cells derived from Cor_93_ TCR-transgenic mice and after 24 hours infected with 10^7^ (acute self-limiting) or 10^8^ pfu (chronic) of adenoviral vector (Ad-HBV-Luc). Liver-associated lymphocytes were isolated after 15 (acute) or 30 days (chronic) and subsequently sorted for Cor_93_-specific CD8^+^ T cells. Single-cell RNA sequencing was performed on 3000 Cor_93_-specific cells per mouse. (B) UMAP projection showing Cor_93_-specific CD8^+^ T cells coloured by condition (C) and cluster identity. (D) Ratio of the number of cells in each cluster in acute and chronic samples. (E) Densities of gene expression levels are visualised by Nebulosa for selected genes and (F) Eno1. (G) GSEA plots comparing the differentially expressed genes (DEGs) for cluster 1 (C1) versus cluster 4 (C4) with the signatures reported in Utzschneider *et al.* 25 (Exhaustion, Exhaustion memory-like and Memory signatures) and the DEGs of acute versus chronic conditions compared with the signatures published in Bengsch *et al.* 26 (Upregulated and downregulated epigenomic exhaustion signatures). Coloured arrows indicate the cluster or condition in which the gene set is enriched. (H) Enolase 1 expression in acute versus chronic setting and cluster 1–4 across conditions. (I) Pathway scores of glycolysis, TCA cycle and oxidative phosphorylation of acute versus chronic setting and among clusters. Mann-Whitney test was performed in (H–I). P values are indicated (p*<0.05, p**<0.01, p***<0.005, p****<0.001). HBV, hepatitis C virus.

Statistical analyses were performed using GraphPad Prism V.9 (GraphPad Prism Software, USA). Normal (Gaussian) distribution of the data was tested using the D’Agostino and Pearson test. Normally distributed data sets (alpha=0.05) were analysed using parametric statistical tests and not normally distributed data sets were analysed using non-parametric statistics. For the comparison of two groups (un)paired t-test, Mann-Whitney U test or Wilcoxon test was used. The comparison of more than two groups was statistically tested using analysis of variance (ANOVA), Kruskal-Wallis test or Friedman test. Tests used in figures 1–7 and online supplemental figures 1–5 were: Unpaired t-test: figure 1C,F and G (centre and right) and online supplemental figure 3F (MTDR, MTDR/MTG) and online supplemental figure 3G (left). Paired t-test: figure 3A (right), figure 4E, figure 6C, online supplemental figure 4H (MTDR/MTG), online supplemental figure 4J, online supplemental figure 5H (left) and I. Wilcoxon test: figure 3A (left), figure 3B,C, figure 4A–C and G–K, figure 6G,I and K, online supplemental figure 4H (MTG, MTDR, MTG^+^MTDR^-^), online supplemental figure 4I,K-M and online supplemental figure 5H (right). Mann-Whitney test: figure 1A,B,D,E and G (left), figure 5B–D, figure 7H,I, online supplemental figure 1C–E, online supplemental figure 2A,B, online supplemental figure 3F (MTG, MitoSox), online supplemental figure 3G (right) and online supplemental figure 3H. ANOVA: figure 6L (right), online supplemental figure 5B,E. Kruskal-Wallis test: figure 2D–I, figure 5A, figure 6B, online supplemental figure 1G,H and online supplemental figure 4B. Friedman test: figure 6H,J and L (left) and online supplemental figure 5C,F. Pearson correlation: online supplemental figure 2G (right). Spearman correlation: figure 3D, figure 4D,F, figure 5E–F, figure 6D–F, online supplemental figure 2C–F, G (left), H–L, online supplemental figure 3B–D and online supplemental figure 4C–G. Statistical tests used are depicted in the figure legends (p*>0.05; p**>0.01; p***>0.001; p****>0.0001). Please see online supplemental methods for additional methods used.

10.1136/gutjnl-2022-328734.supp2 Supplementary data

10.1136/gutjnl-2022-328734.supp3 Supplementary data

## Results

### Heterogeneity of exhaustion profiles indicates bias towards severe exhaustion of HCV-specific CD8^+^ T cells

We set out to understand how metabolic profiles of HBV-specific and HCV-specific CD8^+^ T cell responses in chronic infection are connected to their exhaustion profiles. Virus-specific CD8^+^ T cells were identified by *ex vivo* tetramer staining which revealed a higher frequency of HBV core_18-27_-specific compared with HCV NS3_1073-1081_-specific, NS3_1406-1415_-specific and NS5B_2594-2602_-specific CD8^+^ T cells (figure 1A). HCV-infected patients had more variable liver inflammation with more severe hepatitis identified by transaminase elevation compared with our cohort of HBeAg-negative cHBV patients, as expected (online supplemental tables 1 and 2).^6 9^ We characterised the expression of exhaustion markers that inform about early differentiated ‘progenitor’ exhausted T cells with homeostatic properties and more severely exhausted T cell populations.^7 18^ PD-1 expression was shared by HBV-specific and HCV-specific CD8^+^ T cells but expression of the interleukin 7 receptor α-chain (CD127) was significantly reduced in HCV-specific CD8^+^ T cells, resulting in an enrichment of the PD-1^+^CD127^+^ subset in HBV core_18-27_-specific CD8^+^ T cells (figure 1B–D). Of note, HCV-specific CD8^+^ T cells also expressed higher levels of CD38, CD39 and CD57, indicating stronger activation and a more severely exhausted phenotype, while no major differences in TIGIT, CD27, CD28 and CD122 expression were observed (figure 1E–G). Together, these data indicate significant heterogeneity of the exhaustion profiles of HBV core_18-27_-specific and HCV NS3_1073-1081_-specific, NS3_1406-1415_-specific and NS5B_2594-2602_-specific CD8^+^ T cells in our cohort.

### Hepatitis virus-specific CD8^+^ T cells exhibit different metabolic programmes

Our exhaustion analysis indicated that the comparison of HBV core_18-27_-specific CD8^+^ T cells and HCV NS3_1073-1081_-specific, NS3_1406-1415_-specific and NS5B_2594-2602_-specific CD8^+^ T cells also reflected a comparison between mild and severe exhaustion programmes. The comparison of HBV-specific and HCV-specific CD8^+^ T cell responses thus may not only reflect differences in viral aetiology but also serves as a model to study differences in T cell exhaustion programmes. To understand the metabolic profiles of virus-specific CD8^+^ T cells in chronic HBV and HCV infection, we first performed transcriptome analysis of sorted virus-specific CD8^+^ T cells in untreated chronic infection. Interestingly, gene set enrichment analysis (GSEA) of KEGG metabolic pathways indicated significant differences in the metabolic programmes of mildly exhausted HBV-specific compared with severely exhausted HCV-specific CD8^+^ T cells (figure 2A). In particular, the gene sets for OXPHOS and citric acid cycle (TCA cycle) were significantly enriched in HBV-specific CD8^+^ T cells, while expression of genes involved in glycolysis had a non-significant trend towards higher expression in HCV-specific CD8^+^ T cells (figure 2B). These transcriptional analyses suggested differences in energy metabolism of HBV-specific and HCV-specific CD8^+^ T cells. We thus explored the functional consequences of these altered transcriptomes and functionally interrogated T cell metabolism using metabolism-directed flow cytometry. Glucose uptake was measured using 2-NBDG assay. HBV-specific and HCV-specific CD8^+^ T cells incorporated more 2-NBDG than naïve T cells, in line with higher bioenergetic requirements of activated antigen-specific CD8^+^ T cells (figure 2C,D).

We next assessed the mitochondrial properties of virus-specific CD8^+^ T cells using several mitochondrial dyes that differ in their ability to stain mitochondria depending on their membrane potential. TMRE staining as a direct correlate of ΔΨm electronegativity was significantly higher in HBV-specific compared with HCV-specific CD8^+^ T cells, indicating elevated mitochondrial membrane potential (figure 2E). In contrast, elevated mitochondrial mass, as indicated by MitoTracker Green (MTG) staining was observed in HCV-specific CD8^+^ T cells despite similar total cellular uptake of the MitoTracker Deep Red (MTDR) that correlates with mass and potential (figure 2F,G). The MTDR/MTG ratio (informing about relative mitochondrial polarisation as another estimate of mitochondrial membrane potential) was reduced in HCV-specific CD8^+^ T cells, fitting to the TMRE results (figure 2H). We also observed higher mitochondrial ROS in HBV-specific and HCV-specific CD8^+^ T cells compared with naïve T cells, however, there was a clear enrichment of ROS in HCV-specific compared with HBV-specific CD8^+^ T cells (figure 2I). These data inform about a pronounced alteration of mitochondrial function in HCV NS3_1073-1081_-specific, NS3_1406-1415_-specific and NS5B_2594-2602_-specific CD8^+^ T cells and, together, indicated significant heterogeneity in the metabolic programming of hepatitis virus-specific CD8^+^ T cells during chronic infection.

To understand if these surrogates of mitochondrial function translate into a differential ability to respire, we performed Seahorse analysis of (n=16) *in vitro* expanded HBV-specific and HCV-specific CD8^+^ T cell lines and control cell lines. We observed significant metabolic heterogeneity across these cell lines (online supplemental figure 1A,B). Interestingly, HBV-specific CD8^+^ T cells had a higher spare respiratory capacity compared with HCV-specific CD8^+^ T cell lines (online supplemental figure 1C). In glucose-starved flux assays, there was a trend towards higher glycolytic capacity in HBV-specific CD8^+^ T cell lines (online supplemental figure 1D). The difference in the glycolytic capacity was significant in the flux analysis of non-starved cells (online supplemental figure 1E). These data suggest that a higher relative metabolic fitness of HBV-specific CD8^+^ T cells can be observed *in vitro*, correlating to the *ex vivo* profiling. The ability of HBV-specific CD8^+^ T cells to respond to oligomycin-mediated inhibition of ATP synthase with increased ECAR indicates higher metabolic flexibility to alternatively use glycolysis for ATP production (online supplemental figure 1F). This is in contrast to HCV-specific CD8^+^ T cells, which in some cell lines could not increase their ECAR at all, despite effects on OCR (online supplemental figure 1G,H). In summary, these experiments illustrate significant metabolic heterogeneity in HBV-specific and HCV-specific CD8^+^ T cells with a bias to more significant mitochondrial impairment in severely exhausted HCV-specific CD8^+^ T cells.

### The mitochondrial profile of HBV-specific and HCV-specific CD8^+^ T cells is associated with their differential PD-1/CD127 expression

We next examined the extent to which the mitochondrial polarisation depends on the exhausted subpopulations in cHBV and cHCV infection. Analysis of mitochondrial mass, potential and polarisation between PD-1^+^CD127^+^ and PD-1^+^CD127^-^ virus-specific CD8^+^ T cells in cHBV and cHCV infection revealed significantly reduced mitochondrial polarisation in the more exhausted PD-1^+^CD127^-^ subset, independent of aetiology of viral infection (figure 3A–C). In line with this observation, linear regression analysis also revealed a direct correlation of mitochondrial polarisation and the PD1/CD127 phenotype. Mitochondrial polarisation (MTDR/MTG) inversely correlated with the PD-1^+^CD127^-^ subset frequency, in contrast to a positive correlation with the mildly exhausted subset (PD-1^+^CD127^+^) in HBV-specific and HCV-specific CD8^+^ T cells (figure 3D). These analyses demonstrate a connection of the mitochondrial polarisation with the distribution of different exhaustion subsets of HBV core_18-27_-specific and HCV NS3_1073-1081_-specific, NS3_1406-1415_-specific and NS5B_2594-2602_-specific CD8^+^ T cells and suggest that the metabolic differences are connected to the differentiation of exhausted subsets rather than differences inherent to the different hepatitis viruses.

### Antigen clearance improves mitochondrial fitness of HCV-specific CD8^+^ T cells linked to accumulation of PD-1^+^CD127^+^ populations

Since we had identified severely impaired mitochondrial metabolism in exhausted HCV-specific CD8^+^ T cells associated with a terminally exhausted phenotype (PD-1^+^CD127^−^) during chronic infection that is associated with high levels of antigen stimulation and liver inflammation, we sought to understand if direct-acting antiviral (DAA) therapy would reprogramme HCV-specific CD8^+^ T cell metabolism. Previous studies had shown controversial results on a bulk virus-specific analytic level after overnight culture.^11 22^ Therefore, we performed a longitudinal metabolic analysis of HCV-specific CD8^+^ T cells *ex vivo* in cHCV patients treated with DAA therapy. Interestingly, *ex vivo* analysis identified a significant increase of virus-specific cells at week 2 after therapy initiation (figure 4A), suggesting an augmented virus-specific response, that occurred when transaminase levels significantly declined (figure 4B).

Metabolic analysis revealed an increase in the polarisation of mitochondria after 2 weeks of therapy (figure 4C). Interestingly, we observed a negative association between mitochondrial fitness and ALT levels (figure 4D). Thus, our *ex vivo* analyses indicated a metabolic response to DAA therapy. We next wondered if the improved mitochondrial fitness was associated with a change in the distribution of exhaustion subsets. Indeed, our monitoring showed an enrichment of PD-1^+^CD127^+^ cells during DAA therapy (figure 4E). Clearly, the increased frequency of the PD-1^+^CD127^+^ subset correlated with higher mitochondrial polarisation (figure 4F). We, thus, wondered if DAA therapy augmented the metabolism in severely exhausted cells or if the increase in mitochondrial metabolism is due to an accumulation of the metabolically fit subset. We did not observe significant changes in mitochondrial metabolism in the PD-1^+^CD127^+^ cells during therapy (figure 4G–I). In contrast, there was an increase in polarisation in the remaining severely exhausted PD-1^+^CD127^−^ cells during therapy (figure 4G–I). The augmentation of mitochondrial metabolism in the severely exhausted cells compared with the PD-1^+^CD127^+^ subset resulted in a similar mitochondrial polarisation of the remaining PD-1^+^CD127^−^ cells at week 2 (figure 4J,K). In sum, our data link the enhanced metabolic fitness of HCV-specific CD8^+^ T cells after therapy to an expansion of the PD-1^+^CD127^+^ population and improvement of the metabolic fitness of PD-1^+^CD127^−^ cells.

### The metabolic profile of HBV core_18-27_-specific and polymerase_455-463_ -specific CD8^+^ T cell responses significantly differs and correlates with their exhaustion phenotype

In chronic HBV infection, *ex vivo* HBV-specific CD8^+^ tetramer responses are preferentially identified in HBeAg-negative infection.^6^ Comparison of immunodominant epitope responses recently revealed phenotypic and functional differences between HBV core_18-27_-specific and polymerase_455-463_-specific CD8^+^ T cells in patients despite similar low viral loads and inflammation.^9^ We, thus, wondered if these responses differed in their metabolic profile. Interestingly, HBV polymerase_455-463_-specific CD8^+^ T cells had significantly reduced mitochondrial polarisation and increased mitochondrial ROS production compared with HBV core_18-27_-specific CD8^+^ T cells (figure 5A), resembling the metabolic programme enriched in severely exhausted T cells and typical for HCV infection. Consistent with our previous analysis, HBV polymerase_455-463_-specific CD8^+^ T cells had reduced frequencies of the PD1^+^CD127^+^ subset compared with HBV core_18-27_-specific CD8^+^ T cells (figure 5B) and higher expression of severe exhaustion-associated markers CD38, CD39, CD57 and reduced expression of CD28 (figure 5C,D). These data indicate that HBV polymerase_455-463_-specific CD8^+^ T cells have a more severe mitochondrial dysfunction that is connected to their exhaustion programme. Overall these data are in line with the connection of metabolism to exhaustion state also observed in chronic HCV infection.

### Activation and metabolism of HBV polymerase_455-463_-specific CD8^+^ T cell responses correlate with qHBsAg levels

Since the differences in metabolism and exhaustion programme of HBV-specific responses could not be explained by the degree of liver inflammation, we wondered if they were connected to antigen levels. We thus determined qHBsAg levels and performed correlation analyses. Linear regression analysis revealed a correlation between CD39 expression, mitochondrial polarisation and quantitative HBsAg (qHBsAg) levels for HBV polymerase_455-463_-specific responses, while no such correlation was found for HBV core_18-27_-specific CD8^+^ T cell responses (figure 5E,F). This suggests that antigen recognition drives the metabolic and exhaustion programme in polymerase_455-463_-specific CD8^+^ T cell responses but that this differs in HBV core_18-27_-specific CD8^+^ T cell responses.

### Increased transaminase levels correlate with mitochondrial depolarisation in PD-1^+^CD127^-^ virus-specific CD8^+^ T cells in chronic HCV infection

The differentiation of exhausted T cells is strongly influenced by viral antigen but is also subject to inflammatory cues. In DAA therapy during HCV infection, we had observed a negative correlation of mitochondrial polarisation with ALT (figure 4D), however, the control of viral replication and resolution of liver inflammation are intertwined. Therefore, we next investigated the connection of the metabolic T cell phenotype with the clinical activity in chronic HBV and HCV infection. Patients with cHCV infection displayed a diverse range of liver inflammation as indicated by aspartate aminotransferase (AST) and alanine aminotransferase (ALT) enzyme activity and viral loads, while in our cohort, HBV patients had milder hepatic inflammation (online supplemental figure 2A,B, online supplemental tables 1 and 2). We observed an inverse relationship of liver transaminases and mitochondrial polarisation and activation/exhaustion state of HCV-specific CD8^+^ T cells but not in HBV infection (online supplemental figure 2C–L). There was no clear correlation with viral load as measured by PCR, however, it is unclear if that readout is a good measure of T cell antigen recognition (online supplemental figure 3A–D). There was no direct correlation of metabolic T cell features and transaminase levels in HBeAg-negative chronic infection (ENCI) and HBeAg-negative chronic hepatitis B (ENCHB) patients (online supplemental figure 3E–H). These data show a link between the mitochondrial impairment of more severely exhausted virus-specific CD8^+^ T cells and the degree of hepatic inflammation in cHCV infection.

### Enolase expression is reduced in severely exhausted CD8^+^ T cells and correlates with mitochondrial membrane potential

Our data demonstrate a prominent role for mitochondrial depolarisation in severely exhausted CD8^+^ T cells. However, this depolarisation occurred despite high glucose uptake by HCV-specific CD8^+^ T cells (figure 2D) and despite reduced utilisation of glucose for glycolysis in the metabolic flux analysis (online supplemental figure 1). We, therefore, sought to understand whether glycolytic flux was altered due to dysregulation of enzymes required for glycolysis (figure 6A). MRNA analysis identified variable expression of glycolytic genes between HBV-specific, HCV-specific and CMV-specific CD8^+^ T cells (online supplemental figure 4A). We focused on the analysis of *Eno1* (encoding enolase 1) (online supplemental figure 4B). Intracellular staining for ENO1 revealed highest expression in naïve CD8^+^ T cells, and reduced expression in HCV NS3_1073-1081_-specific and NS3_1406-1415_-specific CD8^+^ T cells compared with HBV core_18-27_-specific and polymerase_455-463_-specific CD8^+^ T cells (figure 6B).

We next investigated the relationship between exhausted T cell subsets and ENO1 expression. ENO1 was significantly more highly expressed in the metabolically fit PD-1^+^CD127^+^ subset compared to PD-1^+^CD127^-^ virus-specific CD8^+^ T cells in chronic HBV and HCV infection (figure 6C). ENO1 expression was inversely correlated with the expression of activation/exhaustion marker CD38, but not with CD39 (figure 6D, online supplemental figure 4C). However, ENO1 expression positively correlated with mitochondrial polarisation (figure 6E), and there was a trend towards decreased mitochondrial ROS with increasing ENO1 expression (online supplemental figure 4D) in line with a model in which enolase levels more directly impact mitochondrial polarisation and low levels of enolase downstream predispose the cell to mitochondrial ROS production. Interestingly, elevated ALT levels also inversely correlated with ENO1, whereas AST levels, viral loads and qHBsAg did not (figure 6F, online supplemental figure 4E-G). These data indicated that ENO1 was expressed by T cells with less severe exhaustion and better mitochondrial fitness, and provoked the hypothesis that ENO1 could support the mitochondrial function of virus-specific CD8^+^ T cells, counteracting exhaustion.

### Enolase enzymatic activity controls virus-specific CD8^+^ T cell metabolism by regulating glycolytic flux

To investigate the functional role of ENO1, we performed extracellular flux analysis of expanded HBV-specific and HCV-specific CD8^+^ T cells and analysed their glycolytic capacity in the presence of enolase inhibitor^23^ (online supplemental figure 5A–G) and/or after addition of the downstream metabolite pyruvate. Metabolic flux analysis showed that enolase inhibition reduced glycolysis in HBV-specific and HCV-specific CD8^+^ T cells (online supplemental figure 5B,E). However, the drop in glycolysis could be overcome by simultaneous injection of the downstream metabolite pyruvate (online supplemental figure 5B,E). Interestingly, enolase inhibition did not cause changes in oxygen consumption, but pyruvate addition caused an immediate small drop in OCR, suggesting that the effects on glycolysis were dominant (online supplemental figure 5C,F). Comparison of the effect of enolase inhibition on glycolysis showed that HBV-specific CD8^+^ T cells were enriched in strong responders to enolase inhibition compared with HCV-specific CD8^+^ T cells, suggesting a stronger role of enolase for the glycolytic flux in these cells. This notion is in line with the higher expression levels of enolase observed in HBV-specific CD8^+^ T cells (figure 6B). The dominant effect of enolase inhibition on glycolysis was also observed when cells were exposed to inhibitor pretreatment before the extracellular flux analysis (online supplemental figure 5H–I). Thus, enolase can act as a glycolytic checkpoint in HBV-specific and HCV-specific CD8^+^ T cells and pyruvate can bypass enolase inhibition for enhanced glycolysis.

### Enolase 1 inhibition can drive mitochondrial depolarisation in primary human CD8^+^ T cells

Since short-term effects of enolase inhibition on respiration in *in vitro* metabolic flux assays were limited, but the correlations of enolase and mitochondrial respiration were strong in *ex vivo* analysis, we next asked if prolonged regulation of glycolysis via ENO1 would also impact on mitochondrial polarisation of freshly isolated primary human CD8^+^ T cells and treated CD8^+^ T cells from healthy individuals overnight with an enolase-specific inhibitor. We observed that ENO1 inhibition resulted in significantly reduced mitochondrial polarisation, more depolarised mitochondria and trends towards reduced cytokine production (online supplemental figure 4H,I). Thus, ENO1 inhibition resulted in a mitochondrial phenotype typical for severely exhausted virus-specific CD8^+^ T cells.

### Enolase enzymatic activity controls virus-specific CD8^+^ T cell effector function

Due to the prominent role of glycolysis for T cell effector function, we next wondered if enolase would represent a metabolic regulator of exhausted T cell function in virus-specific CD8^+^ T cells. We, therefore, examined cytokine production of HBV core_18-27_-specific and HCV NS3_1073-1081_-specific, NS3_1406-1415_-specific and NS5B_2594-2602_-specific CD8^+^ T cells after overnight treatment with AP-III-a4 hydrochloride or in combination with pyruvate. Treatment did not affect viability (online supplemental figure 4J). After treatment with the ENO1 inhibitor, HBV-specific and HCV-specific CD8^+^ T cells showed a significant drop in IFN-γ production (figure 6G). In contrast, the addition of pyruvate enhanced cytokine production (figure 6H–L, online supplemental figure 4K-M). The effects of enolase inhibition on IFN-γ production were stronger than on TNF-α production, however, pyruvate supplementation also enhanced the production of this cytokine, in particular in HCV-specific CD8^+^ T cells (figure 6J).

### Enolase 1 expression is reduced in exhausted versus memory-like CD8^+^ T cells in HBV infection in mice

To understand the role of differential ENO1 expression in hepatitis virus-specific CD8^+^ T cell exhaustion in the same virus model and intrahepatic environment, and to understand differences between acute and chronic infection, we used a recently developed mouse model of acute or chronic HBV infection.^24^ In this model, C57Bl/6 mice were infected with different doses of an adenoviral vector encoding for a 1.3-overlength HBV genome (Ad-HBV-Luc) after transfer of naïve HBV Cor_93_-specific CD8^+^ T cells. Depending on the dose of Ad-HBV-Luc administered, mice develop either acute self-limiting (10^7^ pfu) or chronic HBV infection (10^8^ pfu) (figure 7A). At 15 dpi and 30 dpi, respectively, liver-associated lymphocytes were isolated and Cor_93_-specific CD8^+^ T cells were analysed by single-cell RNA-seq. UMAP analysis identified different antigen-specific T cell transcriptomes in acute and chronic infection (figure 7B). Clustering analysis revealed four separate clusters with differential expression of exhaustion markers (figure 7C). HBV-specific CD8^+^ T cells from mice with acute HBV infection were predominantly enriched in cluster 1 (C1). Chronic HBV-specific CD8^+^ T cells were enriched in C4 (figure 7D). Markers associated with severe exhaustion, such as *Pdcd1*, *Cd38*, *Cd39*, *Tox* and *Fli1* showed increased expression in C4 (figure 7E). In contrast, C1 highly expressed *Il7r*, *Tcf7*, *Slamf6* and *Cxcr5* that indicate homeostatic and memory-like characteristics. Interestingly, *Eno1* expression was significantly higher in acute compared with chronic HBV-specific CD8^+^ T cells, indicating a loss of enolase expression during chronic infection (figure 7F). GSEA of exhaustion signatures revealed a significant enrichment of exhaustion genes in HBV-specific CD8^+^ T cells isolated from chronic condition, as expected. Moreover, direct comparison of C1 versus C4 showed a significant enrichment of exhaustion-associated gene expression in C4 and an enrichment of memory-associated features in C1 (figure 7G).^25 26^ Moreover, C1 was significantly enriched for *Eno1* expression compared with other clusters (figure 7H). These results are in line with our previous findings on ENO1 expression in less severely exhausted virus-specific CD8^+^ T cells in cHBV and cHCV patients (figure 6C). Moreover, we observed significantly elevated glycolytic gene expression in acute versus chronic cells, with C1 exhibiting highest expression of glycolytic genes among all clusters, as well as an enrichment of genes responsible for OXPHOS in acute versus chronic HBV infection. In sum, these data support a role for enolase in metabolically regulating differential exhaustion profiles of intrahepatic virus-specific CD8^+^ T cells (figure 7I).

## Discussion

T cell exhaustion is a prominent feature of virus-specific CD8^+^ T cells in chronic HBV and HCV infection and linked to metabolic changes. Here, by performing a detailed comparison of the metabolic states of HBV core_18-17_-specific and polymerase_455-463_-specific and HCV NS3_1073-1081_-specific, NS3_1406-1415_-specific and NS5B_2594-2602_-specific CD8^+^ T cells from patients with chronic infection, we identified major differences in the metabolism of virus-specific CD8^+^ T cell responses with pronounced mitochondrial dysregulation in severely exhausted PD-1^+^CD127^-^ CD8^+^ T cells enriched in chronic HCV infection. In contrast, higher mitochondrial fitness was observed in PD1^+^CD127^+^ CD8^+^ T cells that have also been termed ‘memory-like’ due to their homeostatic features.^7 25^ Interestingly, the link between metabolism and exhaustion subtype was independent of the viral aetiology. In cHCV infection, the metabolic state also correlated with the extent of liver inflammation. In cHBV infection, we observed different roles for different HBV-specific CD8^+^ T cell responses. In particular, HBV polymerase_455-463_-specific CD8^+^ T cells showed a more severe mitochondrial dysfunction that correlated with viral antigen levels, while HBV core_18-27_-specific CD8^+^ T cell responses were enriched in the PD1^+^CD127^+^ exhaustion phenotype, had relatively intact metabolism but also did not show a clinical correlation. Taken together, these findings suggested a conserved mechanism of metabolic programming in exhausted T cells across viral aetiologies and suggested specific metabolic checkpoints governing different exhaustion states. We identified differential enolase expression as a potential metabolic regulator and speculated if it controls the exhaustion states. Indeed, enolase inhibition resulted in reduced metabolic flux and mitochondrial depolarisation. Bypassing enolase regulation in HBV-specific and HCV-specific CD8^+^ T cells augmented their glycolysis and T cell effector function. In sum, these findings indicate that hepatitis virus-specific CD8^+^ T cells in cHBV and cHCV infection have different exhaustion states due to distinct metabolic programmes. The identification of enolase 1 as a contributing regulator of these exhaustion programmes may provide opportunities for targeted intervention.

Persistent antigen stimulation is a major driver of T cell exhaustion.^27^ DAA therapy in chronic HCV infection causes rapid inhibition of viral replication and served as an *in vivo* model to analyse the effect of tuning viral antigen and associated liver inflammation. Our analysis showed that after 2 weeks of DAA therapy, HCV-specific CD8^+^ T cell metabolism improved with enhanced mitochondrial polarisation. The impact of DAA therapy on T cell metabolism has also been investigated by Aregay *et al.* who, however, did not find a major effect on mitochondrial HCV-specific CD8^+^ T cell metabolism.^11^ In contrast, our DAA therapy results fit to work by Barili *et al.* who also observed improved mitochondrial polarisation in HCV-specific CD8^+^ T cells by focusing on epitope-matched virus-specific CD8^+^ T cell responses.^22^ In these studies, different types of exhaustion subsets were not analysed in detail. In our work, the enhanced mitochondrial fitness after DAA therapy was clearly linked to an accumulation of PD1^+^CD127^+^ virus-specific CD8^+^ T cells. However, our data also suggest that more exhausted subsets experience stronger improvement in metabolism by DAA therapy. Thus, different distributions of exhausted T cell subsets at baseline time points may contribute to the different results found in these earlier studies.^11 22^ Interestingly, while we observed a correlation of the metabolic state of HCV-specific CD8^+^ T cells with liver inflammation that is intertwined with viral replication and antigen recognition, we did not observe such a correlation with liver inflammation in chronic HBV infection. However, in cHBV infection we observed clearly distinct roles for HBV core_18-27_-specific and polymerase_455-463_-specific responses, in line with previous results from phenotypic and functional profiling.^9^ HBV polymerase_455-463_-specific CD8^+^ T cell responses displayed more diverse metabolic and exhaustion states. Importantly, we also observed correlations of activation and mitochondrial dysfunction in HBV polymerase_455-463_-specific CD8^+^ T cell responses with qHBsAg levels in these patients, suggesting that the exhaustion state and the associated metabolism of HBV polymerase_455-463_-specific CD8^+^ T cell response is due to the degree of antigen recognition in cHBV infection. These findings also suggest that antigen is a dominant driver of exhausted T cell metabolism in our cohort, although a contribution of the inflammatory microenvironment, in particular in cHCV infection, cannot be formally excluded.

Glycolysis is key to effector T cell differentiation and function and subject to regulation by immune checkpoints.^14 17 18 28^ Here, in established human chronic infection, we observed high glucose uptake but reduced metabolic activity in severely exhausted CD8^+^ T cells. These cells showed significant mitochondrial disturbances similar to exhausted T cells in LCMV or tumour models.^18 29–31^ It was puzzling that these exhausted T cells also had high glucose uptake despite limited glycolysis and mitochondrial impairment, which prompted us to dissect the glycolytic pathway. Our analysis revealed reduced ENO1 expression in severely exhausted PD-1^+^CD127^-^ CD8^+^ T cells enriched in chronic HCV infection. Inhibition of ENO1 resulted in decreased glycolytic function that could be reversed by downstream pyruvate supplementation. These results are consistent with work that identified reduced enolase activity associated with insufficient effector function in tumour-infiltrating lymphocytes which was reversed after checkpoint therapy.^23^ It also fits to the notion that enolase can act as a metabolic checkpoint, as illustrated by its role in governing the differentiation of regulatory T cells.^32^ Enolase is upstream of the production of PEP, a glycolytic intermediate important for sustaining T cell receptor-mediated Ca^2+^-NFAT signalling and effector functions by repressing sarco/ER Ca^2+^-ATPase (SERCA) activity.^17^ Reduced enolase activity may therefore limit calcium signalling which may in turn explain the reduced T cell function observed after enolase inhibition. Some HCV-specific CD8^+^ T cell responses with low glycolytic activity were metabolically unresponsive to enolase inhibition, suggesting a lack of enolase enzymatic activity in those cells. However, this was a rather extreme observation in our cohort, since most exhausted CD8^+^ T cell responses analysed had residual enolase activity. We also observed reduced mitochondrial polarisation and OXPHOS after overnight ENO1 inhibition, suggesting that enolase provides metabolic substrates for the TCA cycle and its regulation could be involved in throttling metabolic flux upstream of the mitochondrial changes in exhausted T cells. In sum, enolase serves as a metabolic checkpoint of exhausted CD8^+^ T cells in viral hepatitis.

Our data demonstrate that HBV-specific and HCV-specific CD8^+^ T cells exhibit distinct metabolic profiles during chronic infection associated with differences in their exhaustion programmes that are linked to antigen recognition and in cHCV infection, with liver inflammation. We identified the glycolytic enzyme enolase as an upstream metabolic checkpoint contributing to the regulation of the metabolic and functional programmes. Boosting enolase function could represent a novel strategy to counteract reduced effector function of virus-specific CD8^+^ T cells in chronic viral hepatitis.

10.1136/gutjnl-2022-328734.supp4 Supplementary data

## Data Availability

Data are available on reasonable request.
