# Supplementary material for: Enolase represents a metabolic checkpoint controlling the differential exhaustion programmes of hepatitis virus-specific CD8+ T cells
Source: Gut. 2023 Aug 4;72(10):1971–84. doi: 10.1136/gutjnl-2022-328734 (PMC10511960; doi:10.1136/gutjnl-2022-328734)
Supplement: Supplementary data [file gutjnl-2022-328734supp001.pdf]

Supplemental tables

Supplemental table 1: cHBV cohort

Patient ID	Sex	Age (years)	Viral load (IU/ml)	AST (U/l)	ALT (U/l)	HBeAg	HBsAg (IU/ml)	Therapy	Genotype	HLA	Tetramer *ex vivo*
cHBV#1	male	65	<10	26	30	negative	nd	naïve	nd	A*02:01	$core_{18-27}$
cHBV#2	female	59	<10	28	32	negative	42.37	naïve	nd	A*02:01	$core_{18-27}$
cHBV#3	male	34	1531	28	45	negative	19848.26	naïve	D	A*02:01	$core_{18-27}$, $pol_{455-463}$
cHBV#4	male	53	15	54	217	negative	0.37	naïve	nd	A*02:01	$core_{18-27}$
cHBV#5	female	36	1033	19	17	negative	227.3	naïve	nd	A*02:01	$core_{18-27}$
cHBV#6	female	46	<10	16	8	negative	0.62	naïve	nd	A*02:01	$core_{18-27}$
cHBV#7	male	49	649	26	37	negative	nd	naïve	A	A*02:01	$core_{18-27}$, $pol_{455-463}$
cHBV#8	male	53	335	126	93	negative	183.92	naïve	D	A*02:01	$core_{18-27}$
cHBV#9	female	25	574	30	33	negative	nd	naïve	D	A*02:01	$core_{18-27}$, $pol_{455-463}$
cHBV#10	male	55	648	26	28	negative	nd	naïve	A	A*02:01	$core_{18-27}$
cHBV#11	male	32	nd	24	38	negative	nd	naïve	nd	A*02:01	$core_{18-27}$
cHBV#12	male	37	720	nd	37	negative	nd	naïve	D	A*02:01	$core_{18-27}$
cHBV#13	female	59	88	64	78	negative	200.97	naïve	A	A*02:01	$core_{18-27}$
cHBV#14	female	25	534	20	18	negative	1706.51	naïve	D	A*02:01	$core_{18-27}$
cHBV#15	male	24	<10	24	24	6.32	nd	naïve	nd	A*02:01	$pol_{455-463}$
cHBV#16	female	41	515	33	48	negative	7082.27	naïve	D	A*02:01	$core_{18-27}$, $pol_{455-463}$
cHBV#17	female	26	545	27	39	negative	17106.18	naïve	A	A*02:09	$core_{18-27}$
cHBV#18	male	50	25	52	102	nd	0.11	naïve	nd	A*02:01	$core_{18-27}$, $pol_{455-463}$
cHBV#19	male	30	3844	127	191	negative	11044.84	naïve	nd	A*02:02	$core_{18-27}$, $pol_{455-463}$
cHBV#20	male	55	196	37	29	nd	1661.31	naïve	nd	A*02:01	$core_{18-27}$
cHBV#21	male	38	5128	26	29	negative	851.61	naïve	nd	A*02:01	$core_{18-27}$, $pol_{455-463}$
cHBV#22	male	26	53584322	28	51	>120	1401.39	naïve	nd	A*02:01	$core_{18-27}$, $pol_{455-463}$
cHBV#23	female	35	52	77	36	negative	1739.63	naïve	nd	A*02:01	$core_{18-27}$, $pol_{455-463}$
cHBV#24	female	59	92090	48	72	negative	602.86	naïve	nd	A*02:01	$pol_{455-463}$
cHBV#25	female	32	955	26	21	nd	2127.53	naïve	nd	A*02:01	$core_{18-27}$
cHBV#26	female	39	101	23	21	negative	1460.9	naïve	D	A*02:01	$pol_{455-463}$
cHBV#27	male	54	883	22	29	negative	nd	naïve	nd	A*02:01	$core_{18-27}$
cHBV#28	female	21	1901	23	30	negative	12615.76	naïve	D	A*02:01	$core_{18-27}$
cHBV#29	female	40	1655	17	15	nd	555.63	naïve	nd	A*02:01	$core_{18-27}$
cHBV#30	female	36	3214	20	23	negative	6083.86	naïve	D	A*02:01	$core_{18-27}$
cHBV#31	female	28	425	19	18	negative	8508.7	naïve	nd	A*02:01	$core_{18-27}$, $pol_{455-463}$

Supplemental table 2: cHCV cohort

Patient ID	Sex	Age (years)	Viral load (IU/ml)	AST (U/l)	ALT (U/l)	Therapy*	Genotype	HLA	Tetramer *ex vivo*
cHCV#1	male	57	138330	70	102	Ledipasvir/Sofosbuvir	1a	A*02:01	$NS3_{1073-1081}$
cHCV#2	female	76	5301347	31	33	Ledipasvir/Sofosbuvir	1b	A*02:01	$NS3_{1073-1081}$
cHCV#3	male	45	5029509	31	52	Ledipasvir/Sofosbuvir	1a	A*02:01	$NS3_{1406-1415}$
cHCV#4	female	71	4370502	34	30	Ledipasvir/Sofosbuvir	5	A*02:01	$NS3_{1073-1081}$
cHCV#5	male	54	667940	121	100	Ledipasvir/Sofosbuvir/Ribavirin	1a	A*02:01	$NS3_{1073-1081}$
cHCV#6	male	45	2937310	51	89	Sofosbuvir/Daclatasvir	1a	A*02:01	$NS3_{1073-1081}$
cHCV#7	male	50	738876	130	182	Sofosbuvir/Velpatasvir	1a	A*02:01	$NS3_{1073-1081}$
cHCV#8	male	61	3287107	88	130	Ledipasvir/Sofosbuvir	1a	A*02:01	$NS3_{1073-1081}$
cHCV#9	male	50	293903	54	91	Ledipasvir/Sofosbuvir/Ribavirin	4	A*02:01	$NS3_{1073-1081}$
cHCV#10	female	75	9056623	56	54	Glecaprevir/Pibrentasvir	1b	A*02:01	$NS3_{1073-1081}$
cHCV#11	male	55	5822243	25	38	Ledipasvir/Sofosbuvir	1a	A*02:01	$NS3_{1073-1081}$
cHCV#12	female	72	297000	nd	nd	naïve	1b	A*02:01	$NS3_{1073-1081}$
cHCV#13	male	54	1799994	68	111	Glecaprevir/Pibrentasvir	3a	A*02:01	$NS3_{1406-1415}$
cHCV#14	female	64	7332185	60	78	Ledipasvir/Sofosbuvir/Ribavirin	1a	A*02:01	$NS3_{1073-1081}$
cHCV#15	male	35	40256	nd	nd	Ombitasvir/Paritaprevir/Ritonavir/Dasabuvir	1b	A*02:01	$NS3_{1073-1081}$
cHCV#16	female	78	189675	56	64	Glecaprevir/Pibrentasvir	1b	A*02:01	$NS3_{1073-1081}$
cHCV#17	female	65	571421	123	199	Sofosbuvir/Ribavirin	2a	A*02 pos.	$NS5B_{2841-2849}$
cHCV#18	female	35	54244	52	64	Ledipasvir/Sofosbuvir	1b	A*02 pos.	$NS3_{1073-1081}$
cHCV#19	female	48	nd	21	23	naïve	1a	A*02:01	$NS3_{1406-1415}$, $NS5B_{2841-2849}$
cHCV#20	male	41	nd	40	42	Peginterferon/Ribavirin	1a	A*02 pos.	$NS3_{1073-1081}$
cHCV#21	male	55	4586011	41	54	Glecaprevir/Pibrentasvir	1	A*02:01	$NS3_{1073-1081}$, $NS3_{1406-1415}$
cHCV#22	female	27	41555	20	28	Sofosbuvir/Peginterferon/Ribavirin	1a	A*02:01	$NS3_{1073-1081}$
cHCV#23	male	32	70997	56	125	Glecaprevir/Pibrentasvir	3a	A*02:01	$NS3_{1073-1081}$
cHCV#24	female	43	123048	33	41	Peginterferon/Ribavirin	1b	A*02:01	$NS3_{1073-1081}$
cHCV#25	female	61	43842	23	22	naïve	1	A*02:01	$NS3_{1073-1081}$
cHCV#26	male	42	95067	43	88	Peginterferon/Ribavirin	1b	A*02:01	$NS3_{1073-1081}$
cHCV#27	male	39	1466614	51	61	naïve	1a	A*02:01	$NS5B_{2841-2849}$
cHCV#28	male	61	1393356	86	142	Peginterferon/Ribavirin/Boceprevir	1b	A*02:01	$NS3_{1073-1081}$
cHCV#29	male	62	159807	89	128	Peginterferon/Ribavirin	3a	A*02:01	$NS3_{1073-1081}$
cHCV#30	female	51	404833	37	51	naïve	1a	A*02 pos.	$NS3_{1073-1081}$
cHCV#31	male	43	337092	49	60	Sofosbuvir/Velpatasvir	3a	A*02 pos.	$NS5B_{2841-2849}$
cHCV#32	male	70	4726534	56	60	Ledipasvir/Sofosbuvir	1a	A*02 pos.	$NS3_{1073-1081}$
cHCV#33	female	54	2158598	85	57	Ledipasvir/Sofosbuvir	1a	A*02:01	$NS3_{1073-1081}$
cHCV#34	female	57	726936	75	103	Ledipasvir/Sofosbuvir	1b	A*02:01	$NS3_{1073-1081}$
cHCV#35	male	39	514053	114	247	Sofosbuvir/Velpatasvir	3a	A*02:01	$NS5B_{2841-2849}$
cHCV#36	male	44	123843	66	130	Ledipasvir/Sofosbuvir	1a	A*02:01	$NS3_{1073-1081}$
cHCV#37	male	56	3238495	46	45	Ledipasvir/Sofosbuvir	1a	A*02:01	$NS3_{1406-1415}$
cHCV#38	male	38	3288239	88	208	Elbasvir/Grazoprevir	1b	A*02:01	$NS3_{1073-1081}$
cHCV#39	male	46	2325161	42	83	Ledipasvir/Sofosbuvir	1a	A*02:01	$NS3_{1073-1081}$
cHCV#40	male	54	4905790	48	47	Glecaprevir/Pibrentasvir	2b	A*02:01	$NS3_{1073-1081}$
cHCV#41	female	58	620160	47	63	Elbasvir/Grazoprevir	1b	A*02:01	$NS3_{1073-1081}$
cHCV#42	male	54	2030000	107	151	Ledipasvir/Sofosbuvir	1a	A*02:01	$NS5B_{2841-2849}$
cHCV#43	male	32	1123852	48	67	Ledipasvir/Sofosbuvir	1a	A*02:01	$NS3_{1406-1415}$
cHCV#44	male	53	479000	53	73	Ledipasvir/Sofosbuvir	1a	A*02 pos.	$NS3_{1073-1081}$
cHCV#45	male	50	1625676	31	48	Elbasvir/Grazoprevir	1b	A*02:01	$NS3_{1073-1081}$
cHCV#46	female	60	429137	34	44	Ombitasvir/Paritaprevir/Ritonavir/Dasabuvir	1b	A*02:01	$NS3_{1406-1415}$
cHCV#47	male	61	2930792	36	43	Glecaprevir/Pibrentasvir	1a	A*02:01	$NS3_{1406-1415}$
cHCV#48	male	55	2905593	36	49	Glecaprevir/Pibrentasvir	1a	A*02:01	$NS3_{1406-1415}$
cHCV#49	male	45	1952905	62	116	Glecaprevir/Pibrentasvir	1a	A*02:01	$NS3_{1073-1081}$
cHCV#50	female	53	207805	12	nd	Glecaprevir/Pibrentasvir	3a	A*02:01	$NS5B_{2841-2849}$
cHCV#51	female	22	2941144	80	94	Elbasvir/Grazoprevir	1a	A*02:01	$NS3_{1406-1415}$
cHCV#52	female	56	nd	nd	51	naïve	nd	A*02:01	$NS3_{1406-1415}$

*Therapy-naïve cHCV patients were used for all analyses except DAA-based studies (see figure 4).

Supplemental table 3: Antibodies for flow cytometric analysis

Antibody	Conjugate	Clone	Isotype	Dilution	Supplier	Cat no.
anti-CCR7	BUV395	3D12	Rat IgG2a, κ	1:200	BD	740267
anti-CD3	AlexaFluor700	SK7	Mouse IgG1, κ	1:33	BioLegend	344822
anti-CD8	APC	SK1	Mouse IgG1, κ	1:200	BD	345775
anti-CD8	BUV496	RPA-T8	Mouse IgG1, κ	1:100	BD	612942
anti-CD27	PE-Dazzle594	M-T271	Mouse IgG1, κ	1:200	BioLegend	356422
anti-CD28	APC-eFluor780	CD28.2	Mouse IgG1, κ	1:20	eBioscience	47-0289
anti-CD28	PE	B-T3	Mouse IgG2a, κ	1:33	Diaclone	854.222.010
anti-CD38	BV421	HIT2	Mouse IgG1, κ	1:50	BioLegend	303526
anti-CD39	BV650	TU66	Mouse IgG2b, κ	1:33	BD	563681
anti-CD45RA	BUV737	HI100	Mouse IgG2b, κ	1:800	BD	612846
anti-CD57	BV605	QA17A04	Mouse IgG1, κ	1:100	BioLegend	393304
anti-CD122	PE-Cy7	TU27	Mouse IgG1, κ	1:33	BioLegend	339014
anti-CD127	BV510	HIL-7R-M21	Mouse IgG1, κ	1:100	BD	563086
anti-CD127	BV711	HIL-7R-M21	Mouse IgG1, κ	1:20	BD	563165
anti-ENO1	AlexaFluor488	EPR10863(B)	Rabbit IgG	1:200	abcam	ab205871
anti-IFN-γ	FITC	25723.11	Mouse IgG2b, κ	1:8.33	BD	340449
anti-PD-1	BV421	EH12.2H7	Mouse IgG1, κ	1:50	Biolegend	329920
anti-PD-1	BV786	EH12.1	Mouse IgG1, κ	1:33	BD	563789
anti-TIGIT	PerCP-eF710	MBSA43	Mouse IgG1, κ	1:50	eBioscience	46-9500
anti-TNF-α	PE-Cy7	MAb11	Mouse IgG1, κ	1:100	BioLegend	502930
