# Supplementary material for: Enolase represents a metabolic checkpoint controlling the differential exhaustion programmes of hepatitis virus-specific CD8+ T cells
Source: Gut. 2023 Aug 4;72(10):1971–84. doi: 10.1136/gutjnl-2022-328734 (PMC10511960; doi:10.1136/gutjnl-2022-328734)
Supplement: Supplementary data [file gutjnl-2022-328734supp002.pdf]

Supplemental figure 1 (related to figure 2)

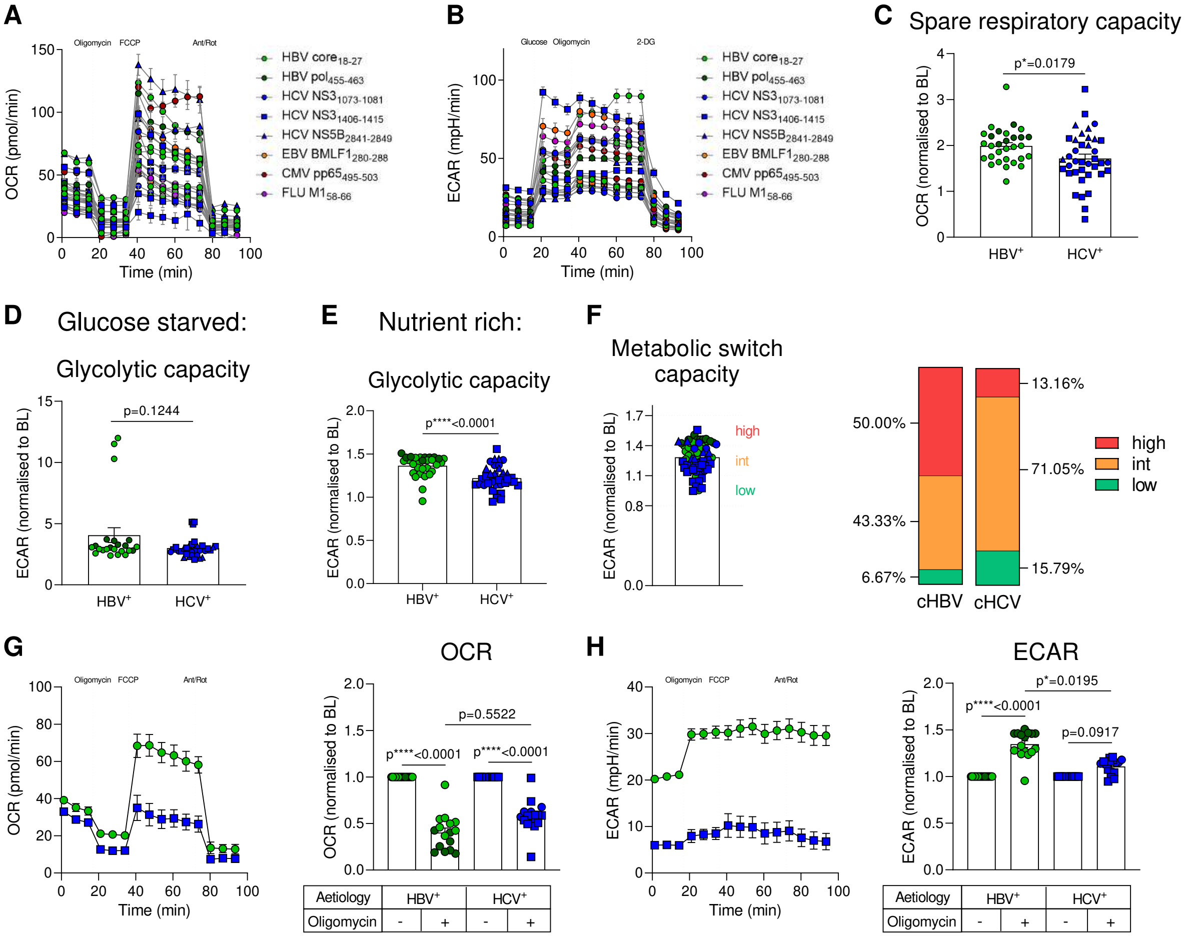

Supplemental figure 2 (related to figure 2)

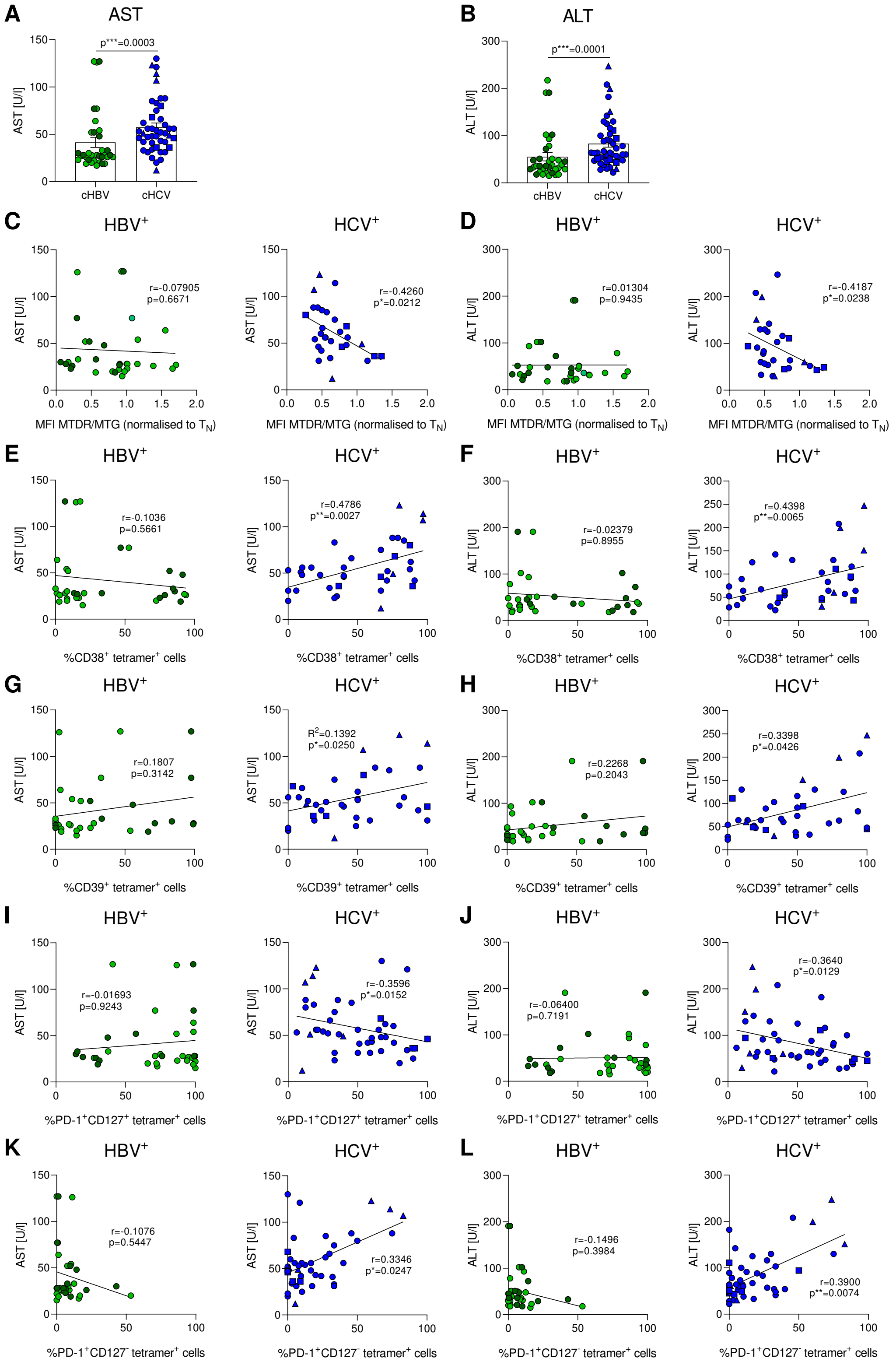

BMJ Publishing Group Limited (BMJ) disclaims all liability and responsibility arising from any reliance placed on this supplemental material which has been supplied by the author(s)

Supplemental figure 3 (related to figure 2)

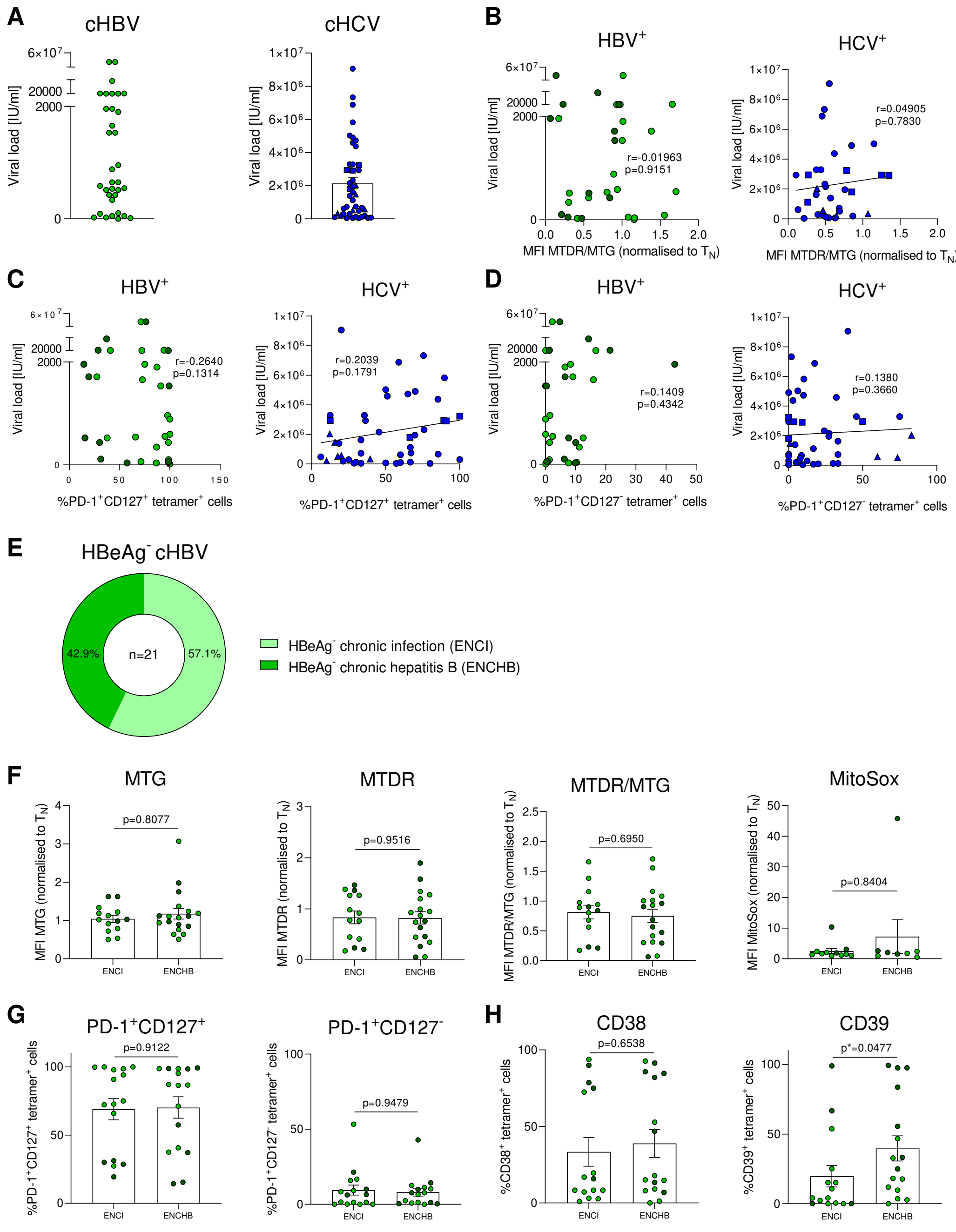

BMJ Publishing Group Limited (BMJ) disclaims all liability and responsibility arising from any reliance placed on this supplemental material which has been supplied by the author(s)

Supplemental figure 4 (related to figure 6)

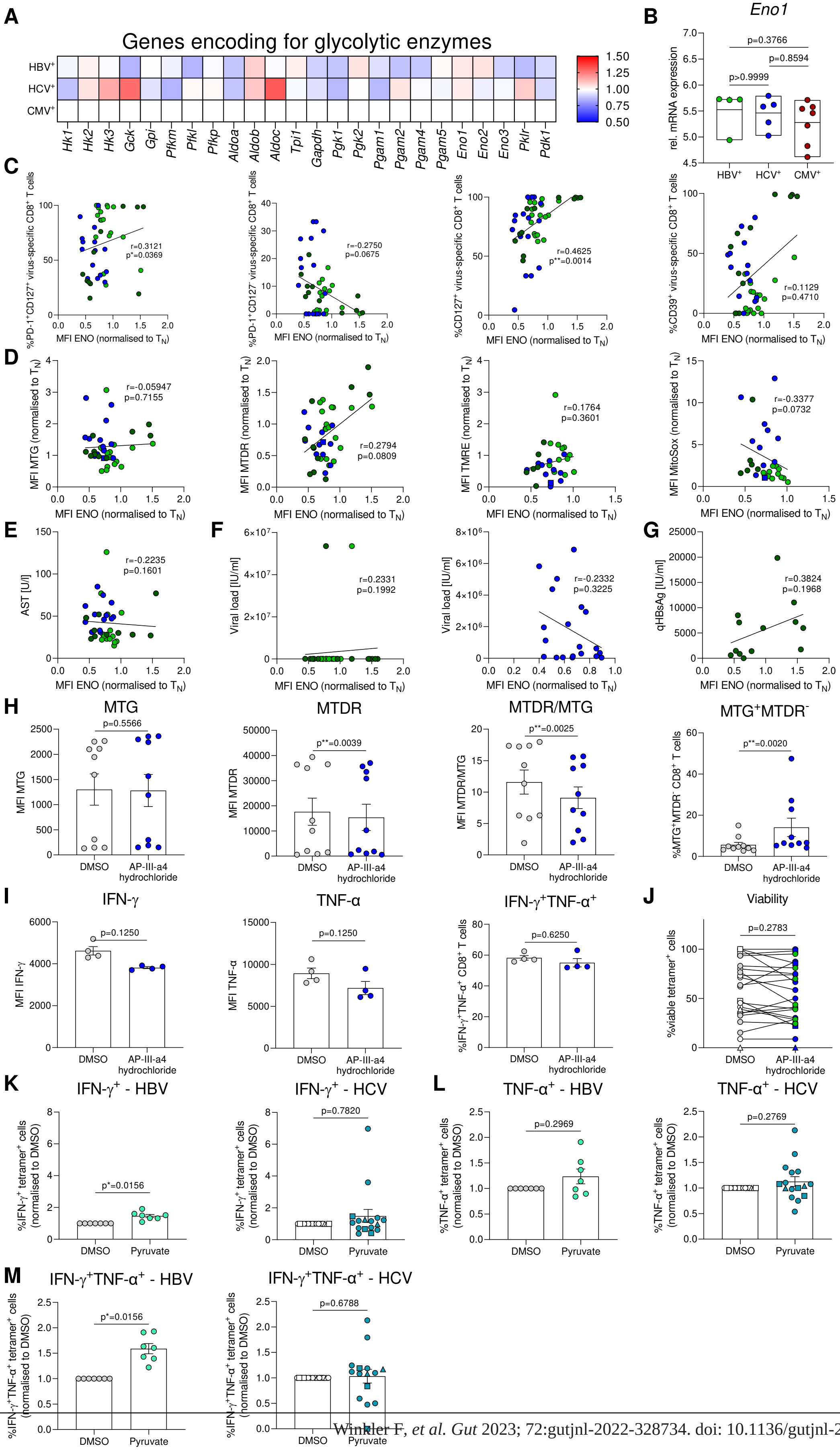

BMJ Publishing Group Limited (BMJ) disclaims all liability and responsibility arising from any reliance placed on this supplemental material which has been supplied by the author(s)

Supplemental figure 5 (related to figure 6)

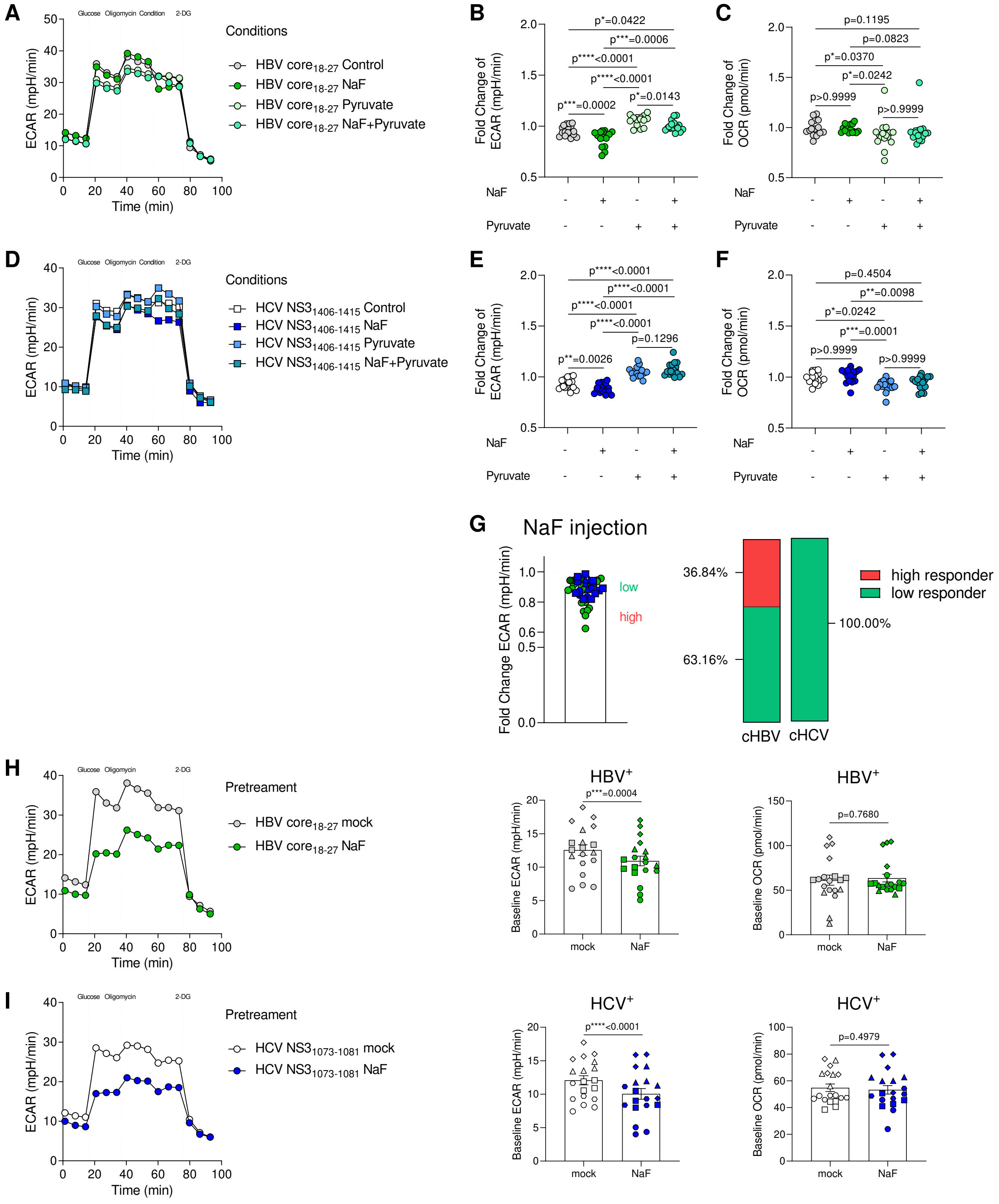

Supplemental figure legends

Supplemental figure 1. *In vitro* expanded HCV-specific CD8$^+$ T cells exhibit diminished spare respiratory capacity and reduced metabolic switch capacity. Seahorse analysis: Quadruplicates of 200 000 expanded HBV- and HCV-specific CD8$^+$ T cells were stimulated with cognate peptide and analysed for oxygen consumption rate (OCR) and extracellular acidification rate (ECAR). **(A)** Mito Stress Test was performed with time-delayed injections of oligomycin (1µM), FCCP (1.5µM), antimycin (1µM) and rotenone (0.1µM). **(B)** In the Glyco Stress Test *in vitro* expanded hepatitis virus-specific CD8$^+$ T cells were exposed to subsequent injections of glucose (10mM), oligomycin (1µM) and 2-DG (50mM). **(C)** Spare respiratory capacity was determined in Mito Stress test experiments as the ratio of maximal respiration (after FCCP injection) to basal respiration. **(D)** Glycolytic capacity was determined in glucose-starved (Glyco Stress test) and **(E)** nutrient-rich (Mito Stress test) conditions as the ratio of maximal acidification rates (after oligomycin injection) to basal acidification rates. **(F)** ECAR after complex V inhibition by oligomycin are shown. Metabolic switch capacities of expanded HBV- and HCV-specific CD8$^+$ T cells were categorised in low, intermediate and high and proportions are visualised. **(G)** OCR and **(H)** ECAR of expanded hepatitis virus-specific CD8$^+$ T cells after oligomycin injection. To ensure appropriate comparison of different batches of expanded virus-specific CD8$^+$ T cells, OCR and ECAR values were normalised to baseline for calculation of spare respiratory capacity and glycolytic capacity. Mann-Whitney test was performed in **(C-E)**. Kruskal-Wallis test was performed in **(G-H)**. Significance is indicated (p*<0.05, p****<0.001). Error bars indicate mean ±SEM.

Supplemental figure 2. Transaminase levels inform exhaustion severity and mitochondrial function of HCV-specific CD8$^+$ T cells. (A-B) Transaminase activity (AST, aspartate aminotransferase; ALT, alanine aminotransferase) was assessed in serum of therapy-naïve cHBV and cHCV patients. **(C-D)** Correlation analyses of AST/ALT levels and mitochondrial polarisation (MTDR/MTG), **(E-F)** frequencies of CD38$^+$, **(G-H)** CD39$^+$, **(I-J)** PD-1$^+$CD127$^+$ and **(K-L)** PD-1$^+$CD127$^-$ virus-specific CD8$^+$ T cells. HBV core$_{18\text{-}27}$ (green) and HCV NS3$_{1073\text{-}1081}$ epitopes (blue) are represented by circles. HCV NS3$_{1406\text{-}1415}$ and HCV NS5B$_{2594\text{-}2602}$ epitopes are visualized by blue squares and triangles, respectively. Mann-Whitney test was performed in **(A-B)**. Spearman r correlation analyses were performed in **(C-F)**, **(G**, left**)** and **(H-L)**. Pearson r correlation analyses were performed in **(G**, right**)**. Significance is indicated (p*<0.05, p**<0.01, p***<0.005). Error bars indicate mean ±SEM.

Supplemental figure 3. HBV-specific CD8$^+$ T cells of HBeAg-negative chronic hepatitis B (ENCHB) patients show increased CD39 expression. (A) Viral loads were determined in therapy-naïve cHBV and cHCV patients by using clinical tests. **(B)** Correlation analyses of

patients' viral loads and mitochondrial polarisation (MTDR/MTG) and **(C)** frequencies of PD-1$^+$CD127$^+$ and **(D)** PD-1$^+$CD127$^-$ subsets of HBV- and HCV-specific CD8$^+$ T cells. **(E)** Serum transaminase level of therapy-naïve HBeAg-negative cHBV patients and **(F)** their categorisation into HBeAg-negative chronic infection (ENCI) and HBeAg-negative chronic hepatitis B (ENCHB) patients. **(G)** Metabolic *ex vivo* staining of HBV-specific CD8$^+$ T cells for mitochondrial mass (MTG), mitochondrial membrane potential (MTDR), mitochondrial polarisation (MTDR/MTG) and mitochondrial ROS (MitoSox) of ENCI and ENCHB patients. Signals of metabolic stainings were normalised to naïve (CCR7$^+$CD45RA$^+$) CD8$^+$ T cells. **(H)** Frequencies of PD-1$^+$CD127$^+$, PD-1$^+$CD127$^-$, **(I)** CD38$^+$ and CD39$^+$ HBV-specific CD8$^+$ T cells in ENCI and ENCHB patients. Patients were classified as ENCI if AST and ALT level were normal and considered ENCHB if either their AST and/ or ALT activity was above the normal range of 10-35 U/l in females and 10-50 U/l in males. HBV core$_{18\text{-}27}$ (green) and HCV NS3$_{1073\text{-}1081}$ epitopes (blue) are represented by circles. HCV NS3$_{1406\text{-}1415}$ and HCV NS5B$_{2594\text{-}2602}$ epitopes are visualised by blue squares and triangles, respectively. Mann-Whitney test was performed in **(E)**, **(G**, MTG and MitoSox**)**, **(H**, right**)** and **(I)**. Unpaired t-test was performed in **(G**, MTDR and MTDR/MTG**)** and **(H**, left**)**. Spearman r correlation analyses were performed in **(B-D)**. Significance is indicated (p*<0.05, p**<0.01, p***<0.005). Error bars indicate mean ±SEM.

Supplemental figure 4. Enolase inhibition induced by AP-III-a4 hydrochloride negatively affects mitochondrial function of CD8$^+$ T cells from healthy individuals. **(A)** Heat map illustrating mRNA expression levels of genes encoding for key enzymes in the glycolytic pathway of therapy-naïve sorted HBV- (n=4) and HCV-specific CD8$^+$ T cells (n=5) normalised to sorted CMV-specific CD8$^+$ T cells (n=7). **(B)** Relative Eno1 mRNA expression of HBV- and HCV-specific CD8$^+$ T cells. **(C)** Correlation analyses of ENO1 expression and frequencies of PD-1$^+$CD127$^+$, PD-1$^+$CD127$^-$, CD127$^+$ and CD39$^+$ hepatitis virus-specific CD8$^+$ T cells **(D)** Correlation analyses of ENO1 expression and mitochondrial mass (MTG), mitochondrial membrane potential (MTDR, TMRE), mitochondrial ROS (MitoSox) and **(E)** serum AST level, **(F)** viral loads of therapy-naïve cHBV and cHCV patients and **(G)** HBsAg of cHBV patients. **(H)** Metabolic *ex vivo* staining of CD8$^+$ T cells isolated from healthy individuals for mitochondrial mass (MTG), mitochondrial membrane potential (MTDR), mitochondrial polarisation (MTDR/MTG) and the frequency of depolarised (MTG$^+$MTDR$^-$) mitochondria after o/n treatment with AP-III-a4 hydrochloride. **(I)** IFN-γ and TNF-α production of PMA- and ionomycin-stimulated CD8$^+$ T cells of healthy donors after o/n incubation with AP-III-a4 hydrochloride. **(J)** Frequencies of viable hepatitis virus-specific CD8$^+$ T cells after o/n exposure to AP-III-a4 hydrochloride. HBV core$_{18\text{-}27}$ (green) and HCV NS3$_{1073\text{-}1081}$ epitopes (blue) are represented by circles. HCV NS3$_{1406\text{-}1415}$ and HCV NS5B$_{2594\text{-}2602}$ epitopes are visualised by blue squares and

triangles, respectively. Kruskal-Wallis test was performed in **(B)**. Spearman r correlation analyses were performed in **(C-G)**. Wilcoxon test was performed in **(H**, MTG, MTDR and $MTG^{+}MTDR^{-}$**)**, **(I)** and **(K-M)**. Paired t-test was performed in **(H**, MTDR/MTG**)** and **(J)**. Significance is indicated (p*<0.05, p**<0.01). Error bars indicate mean ±SEM.

Supplemental figure 5. Enolase represents a metabolic checkpoint restricting the glycolytic flux in hepatitis virus-specific $CD8^{+}$ T cells. (A-F) Glyco Stress test was performed using quadruplicates of 200 000 expanded HBV- and HCV-specific $CD8^{+}$ T cells stimulated with cognate peptide and analysed for oxygen consumption rate (OCR) and extracellular acidification rate (ECAR). To this end, cells received time-delayed injections of glucose (10mM), oligomycin (1µM), sodium fluoride (NaF, 2mM) and/ or sodium pyruvate (2mM) and 2-DG (50mM). **(G)** ECAR values of HBV-and HCV-specific $CD8^{+}$ T cells after NaF injection were discriminated in high and low responders according to their ECAR drop after NaF injection. Frequencies of high and low NaF responders are illustrated. **(H-I)** Expanded hepatitis virus-specific $CD8^{+}$ T cells were exposed to sodium fluoride (2mM) 2h before measurement and basal ECAR and OCR levels were compared to mock-treated HBV- and HCV-specific $CD8^{+}$ T cells. One-way ANOVA was performed in **(B)** and **(E)**. Friedman test was performed in **(C)** and **(F)**. Paired t-test was performed in **(H**, left**)** and **(I)**. Wilcoxon test was performed in **(H**, right**)**. Significance is indicated (p*<0.05, p**<0.01, p***<0.005, p****<0.001). Error bars indicate mean ±SEM.
