# Supplementary material for: Enolase represents a metabolic checkpoint controlling the differential exhaustion programmes of hepatitis virus-specific CD8+ T cells
Source: Gut. 2023 Aug 4;72(10):1971–84. doi: 10.1136/gutjnl-2022-328734 (PMC10511960; doi:10.1136/gutjnl-2022-328734)
Supplement: Supplementary data [file gutjnl-2022-328734supp003.pdf]

Supplemental methods

PBMC isolation

Peripheral blood from therapy-naïve chronically infected hepatitis B- and C patients was collected in EDTA-anticoagulated tubes at the University Medical Center Freiburg, Germany. Venous blood was transferred onto a lymphocyte separation medium gradient (Pancoll, PanBiontech GmBH) following peripheral blood mononuclear cell (PBMC) isolation that have been resuspended in freezing medium containing 80% fetal calf serum (FCS), 10% dimethyl sulfoxide and 10% RPMI1640 and were stored at -80°C until the day of experiment.

Multi-parametric flow cytometry

Prior to staining, PBMCs were thawed in RPMI1640 supplemented with 10% fetal calf serum, 1% penicillin/streptomycin, 1.5% 1M Hepes buffer containing 50U/mL benzonase (Sigma). Staining with a fixable viability dye (APC-eFluor780, 1:200, eBioscience; eFluor506, 1:100, eBioscience) was performed for 5 minutes at RT, followed by a tetramer staining for 15 minutes at RT. Metabolic staining was performed in complete RPMI medium for 20 minutes at 37°C and 5% CO_2. To detect glucose uptake, cells were incubated with the fluorescent glucose analogue 2-[N-(7-nitrobenz-2-oxa-1,3-diazol-4-yl) amino]-2-deoxy-D-glucose (2-NBDG, Thermo Fisher, 200µM). Several metabolic dyes were used to identify different complementary mitochondrial features. Mitochondrial mass was assessed using the MitoTracker Green probe (MTG, Thermo Fisher, 50µM). The mitochondrial mass and potential dependent fluorescent dye MitoTracker Deep Red (MTDR, Thermo Fisher, 10µM) was used in combination with MTG to distinguish polarised from depolarised mitochondria. In addition, the mitochondrial membrane potential was determined by staining with tetramethylrhodamine ethyl ester (TMRE, Biomol, 10nM), which accumulates in mitochondria in inverse proportion to the mitochondrial membrane potential according to the Nernst equation. Mitochondrial superoxides were analysed by exposing of PBMCs to MitoSox Red (MitoSox, Thermo Fisher, 5µM). In a next step, surface staining was performed for 10 minutes at 37°C, 5% CO_2 and 20 minutes at RT. After surface staining, PBMCs were resuspended in PBS containing 1% FCS and protected from light until analysis using FACSCanto II, LSRFortessa with FACSDiva software version 10.6.2 (BD) or CytoFLEX (Beckman Coulter) with CytExpert software version 2.3.0.84. Flow cytometric data were analysed using FlowJo software version 10.6.2 (Treestar). Metabolic stainings were analysed after normalisation of MFI signals to naïve $CD8^+$ T cells from the respective sample (defined as $CCR7^+CD45RA^+$) to address batch variation in metabolic data (**figure 2C**). For the detection of intracellular molecules, the FoxP3/transcription factor staining buffer set (Thermo Fisher) was used according to the

manufacturer's protocol. For sufficient detection of cytokines, PBMCs were stimulated prior to staining with phorbol-12-myristat-13-acetate (PMA, Sigma) and ionomycin (Sigma) in the presence of brefeldin A and monensin (BD) for 5 hours at 37°C and 5% CO_2. After intracellular cytokine staining, PBMCs were fixed in 2% paraformaldehyde (PFA). Antibodies used for flow cytometry are listed in **Supplemental table 3**.

Cell sorting

Freshly isolated PBMCs obtained from chronically HBV- and HCV-infected patients were stained with a fixable viability dye for 5 minutes at RT, the respective tetramer for 15 minutes at RT and a CD8 antibody for 15 minutes at RT, followed by single cell sorting of viable, tetramer$^+$ CD8$^+$ T cells into the sterile 96-well plate with allogenic feeder cells. Cell sorting for the generation of expanded HBV- and HCV-specific CD8$^+$ T cells was performed using the FACSMelody cell sorter (BD).

Microarray analysis

Virus-specific CD8$^+$ T cells from therapy-naïve patients with chronic hepatitis B virus (cHBV) and chronic hepatitis C virus (cHCV) infection and from CMV-positive healthy individuals were identified by flow cytometric staining with HLA-A*02:01-restricted tetramers directed against the following epitopes: HBV core$_{18\text{-}27}$: FLPSDFFPSV (n=4), HCV NS3$_{1073\text{-}1081}$: CINGVCWTV (n=4), HCV NS3$_{1406\text{-}1415}$: KLVALGINAV (n=1) and CMV pp65$_{495\text{-}503}$: NLVPMVATV (n=7). The yield of tetramer-specific CD8$^+$ T cells ranged from 220 to 15 000 cells. The RNeasy mini kit (Qiagen) was used to extract RNA from the flow cytometry sorted cells. Due to the low number of tetramer-positive cells, the RNA was amplified and converted to cDNA using the WT-OvationTM Pico RNA Amplification System (NuGEN, AC Leek, The Netherlands). The resulting single-stranded cDNA was fragmented, labelled using the Encore Biotin Module (NuGEN), and then hybridised to the Affymetrix® Human Genome U219 array plate in the GeneTitan® instrument (Affymetrix, Santa Clara, CA), according to the manufacturer's protocol. The Bioconductor package version 2.12 (working with R version 3.0.1) was used for microarray analysis. Probe annotation was performed using the alternative cdf version 17 based on Entrez Gene, assigning probes to 18 567 unique transcripts. Data pre-processing was performed using the RMA algorithm. Data from three different batches were combined, and a correction for batch effect was applied using the ComBat package. The data are deposited as GEO GSE60552.

Gene set enrichment analysis

Gene set enrichment analysis (GSEA) of metabolic pathways was performed on microarray data from sorted HBV- and HCV-specific $CD8^+$ T cells (deposited in GEO GSE60552) using the GSEA software (version GSEA v4.2.2). Gene sets from the Molecular Signature Database v.7.5.1 [1] including KEGG pathway gene lists obtained from http://www.genome.jp/kegg/pathway.html were used. In addition, the following exhaustion-related gene sets were used: (a) exhaustion (chronic $TCF1^-$), exhaustion memory-like (chronic $TCF1^+$), memory signature [2] and (b) genes up- and downregulated in exhaustion [3]. Normalised enrichment scores (NES) obtained by GSEA were used to compare different pathways of HBV- and HCV-specific $CD8^+$ T cells.

T cell expansion *in vitro*

PBMCs isolated from peripheral blood of HLA-A*02:01-positive cHBV and cHCV patients and healthy donors were stained with HLA-A*02:01-restricted fluorochrome-labelled peptide MHC-I tetramers, viability dye and surface antibodies (CCR7, CD45RA, CD8). In a next step, non-naïve, $tetramer^+CD8^+$ T cells were sorted into a sterile 96-well plate containing 100 µL medium and 200 000 allogeneic feeder cells derived from healthy donors (previously irradiated at 30 Gy for 30 minutes). Sorted single cells were supplemented with 40µg/mL phytohaemagglutinin (PHA) and incubated at 5% CO_2 and 37°C. Every 3-4 days, antigen-specific cells were supplemented with fresh medium (RPMI 1640 containing 10% human serum, 2mM L-glutamine, 100U/mL penicillin and 100µg/mL streptomycin) and 20U/mL IL-2. After 14 days, antigen-specific $CD8^+$ T cells were incubated with freshly isolated allogeneic feeder cells, transferred to a 48-well plate and maintained under the above conditions. After 28 days, the cells were restimulated and transferred to a 24-well plate. After 35 days and visible cell proliferation, expanded $CD8^+$ T cells were flow cytometrically tested for maintenance of antigen specificity and frozen at -80°C until further use. For metabolic flux analysis, antigen-specific $CD8^+$ T cell cultures were thawed and restimulated with allogeneic feeder cells and IL-2 as described above. Every 3-4 days, antigen-specific $CD8^+$ T cells received fresh medium and were supplemented with IL-2. After 14 days, antigen-specific $CD8^+$ T cells were restimulated with allogeneic feeder cells and IL-2. After 21 days, antigen-specific $CD8^+$ T cells were tested for antigen specificity and counted. Quadruplicates of 200 000 expanded virus-specific $CD8^+$ T cells were seeded in a 96-well plate precoated with Cell-Tak (Thermo Fisher Scientific). In total, virus-specific $CD8^+$ T cells were cultured for 8 weeks prior to the Seahorse experiments.

Seahorse extracellular flux analysis

200 000 expanded virus-specific $CD8^+$ T cells were seeded in quadruplicates into a 96-well polystyrene Seahorse plate (Agilent) precoated with Cell-Tak (Thermo Fisher Scientific) and equilibrated for 1 hour at 37°C and 5% CO_2. After equilibration, virus-specific $CD8^+$ T cells were incubated with cognate peptide for 2 hours. Cells were assayed for extracellular acidification rate (mpH/min) and oxygen consumption rate (pmol/min) using the Seahorse XF Cell Mito Stress Test Kit (Agilent) and the Seahorse XF Glycolysis Stress Test Kit. During the Mito Stress Test, cells were exposed to injections of oligomycin (1µM), FCCP (1.5µM), antimycin (1µM) and rotenone (0.1µM). During the Glycolysis Stress Test, cells received injections of glucose (10mM), oligomycin (1µM) and 2-DG (50mM). In addition, *in vitro* expanded antigen-specific $CD8^+$ T cells were injected or pre-incubated with sodium fluoride (2µM) (NaF) (Merck) and/or sodium pyruvate (2mM).

Patient and Public involvement

It was not appropriate or possible to involve patients or the public in the design, or conduct, of our research. Dissemination of the results of this study will be by press release, Twitter and making publications accessible to patients in our outpatient hepatology clinic.

Adenoviral vector

The Ad-HBV-Luc vector developed and reported by Manske *et al*. was used [4].

Mice

C57Bl/6 mice were purchased from Charles River and Cor93 TCR transgenic mice (B6.Cg-Ptprca Pepcb Tg(TcraBC10,TcrbBC10)3Chi/J) were purchased from The Jackson Laboratory. Mice were housed under specific pathogen-free conditions in the central animal facility of the Technical University of Munich, according to the guidelines of the Federation of Laboratory Animal Science Association. Adult male mice (older than 6 weeks) were used in the experiments. Adenoviral vectors were intravenously (i.v.) injected in 100 µL of 0.9% sodium chloride solution. All animal experiments were authorised by permission of the Regierung von Oberbayern (AZ: ROB-55.2.2532.Vet_02-18-90).

Adoptive T cell transfer

Naïve $CD44^{-}CD8^{+}$ T cells were isolated from the spleen and lymph nodes of Cor93 TCR transgenic mice by negative magnetic bead separation (Miltenyi Biotec). Next, 10 000 CD45.1 T cells were intravenously injected into the tail vein in 100 µL PBS.

Isolation of liver-associated lymphocytes

The livers of sacrificed mice were perfused with PBS via the portal vein, finely chopped and passed through a sieve. Liver cells were washed in PBS and then incubated in Gey's balanced salt solution (PAN Biotech,) supplemented with 0.125 U/mg collagenase type 2 (Worthington Biochemical Corporation) for 10 min at 37 °C. After enzymatic digestion, lymphocytes were isolated by Percoll (GE Healthcare) gradient centrifugation. Briefly, cells were resuspended in a 40% Percoll solution and underlaid with an 80% Percoll solution. The isolated lymphocytes were washed in PBS and then used for cell sorting of $Cor93^{+}CD45.1^{+}CD8^{+}$ T cells.

scRNA-Seq preprocessing

ScRNA-Seq preparation was performed using the Chromium Next GEM Single Cell 3´ Kit V3.1 (10x Genomics, Pleasanton, California, US) according to the manufacturer's instructions. Sequencing was performed on a NovaSeq6000 Paired-End S1 Flowcell. scRNA-Seq bioinformatic analysis was performed in R version 4.0.2. The 10x cellranger sequencing pipeline and cell hashing were used to attenuate batch effects and followed the Seurat (4.0.5) vignette to demultiplex the counts information. Doublet cells (with more than one hashtag) were removed. For quality control, we removed cells with the following conditions: a number of UMI less than 300 or more than 30 000; above 5 000 features; mitochondrial reads greater than five percent. Counts were log normalised and scaled. For UMAP projection we used the following parameters n.neighbours = 350, min.dist = 0.01, and 50 dimensions in PCA in the RunUMAP Seurat function. Gene expression densities were visualised using Nebulosa V1.0.2. The glycolysis, TCA cycle and OXPHOS metabolic pathways were obtained from Xiao *et al.* [5]. Cells were scored using AUCell (V1.12.0). For the GSEA analysis we used the signatures of Bengsch *et al*. and Utzschneider *et al*. [2, 3].

Supplemental references

1 Subramanian A, Tamayo P, Mootha VK, Mukherjee S, Ebert BL, Gillette MA*, et al.* Gene set enrichment analysis: a knowledge-based approach for interpreting genome-wide expression profiles. Proc Natl Acad Sci U S A 2005;**102**:15545-50.

2 Utzschneider DT, Charmoy M, Chennupati V, Pousse L, Ferreira DP, Calderon-Copete S*, et al.* T Cell Factor 1-Expressing Memory-like CD8(+) T Cells Sustain the Immune Response to Chronic Viral Infections. Immunity 2016;**45**:415-27.

3 Bengsch B, Ohtani T, Khan O, Setty M, Manne S, O'Brien S*, et al.* Epigenomic-Guided Mass Cytometry Profiling Reveals Disease-Specific Features of Exhausted CD8 T Cells. Immunity 2018;**48**:1029-45 e5.

4 Manske K, Schneider A, Ko C, Knolle PA, Steiger K, Protzer U*, et al.* In Vivo Bioluminescence Imaging of HBV Replicating Hepatocytes Allows for the Monitoring of Anti-Viral Immunity. Viruses 2021;**13**.

5 Xiao Z, Dai Z, Locasale JW. Metabolic landscape of the tumor microenvironment at single cell resolution. Nature communications 2019;**10**:3763.
