# Supplementary figures and images for: Enolase represents a metabolic checkpoint controlling the differential exhaustion programmes of hepatitis virus-specific CD8+ T cells
Source: Gut. 2023 Aug 4;72(10):1971–84. doi: 10.1136/gutjnl-2022-328734 (PMC10511960; doi:10.1136/gutjnl-2022-328734)

Metabolic features of hepatitis virus-specific CD8<sup>+</sup> T cells

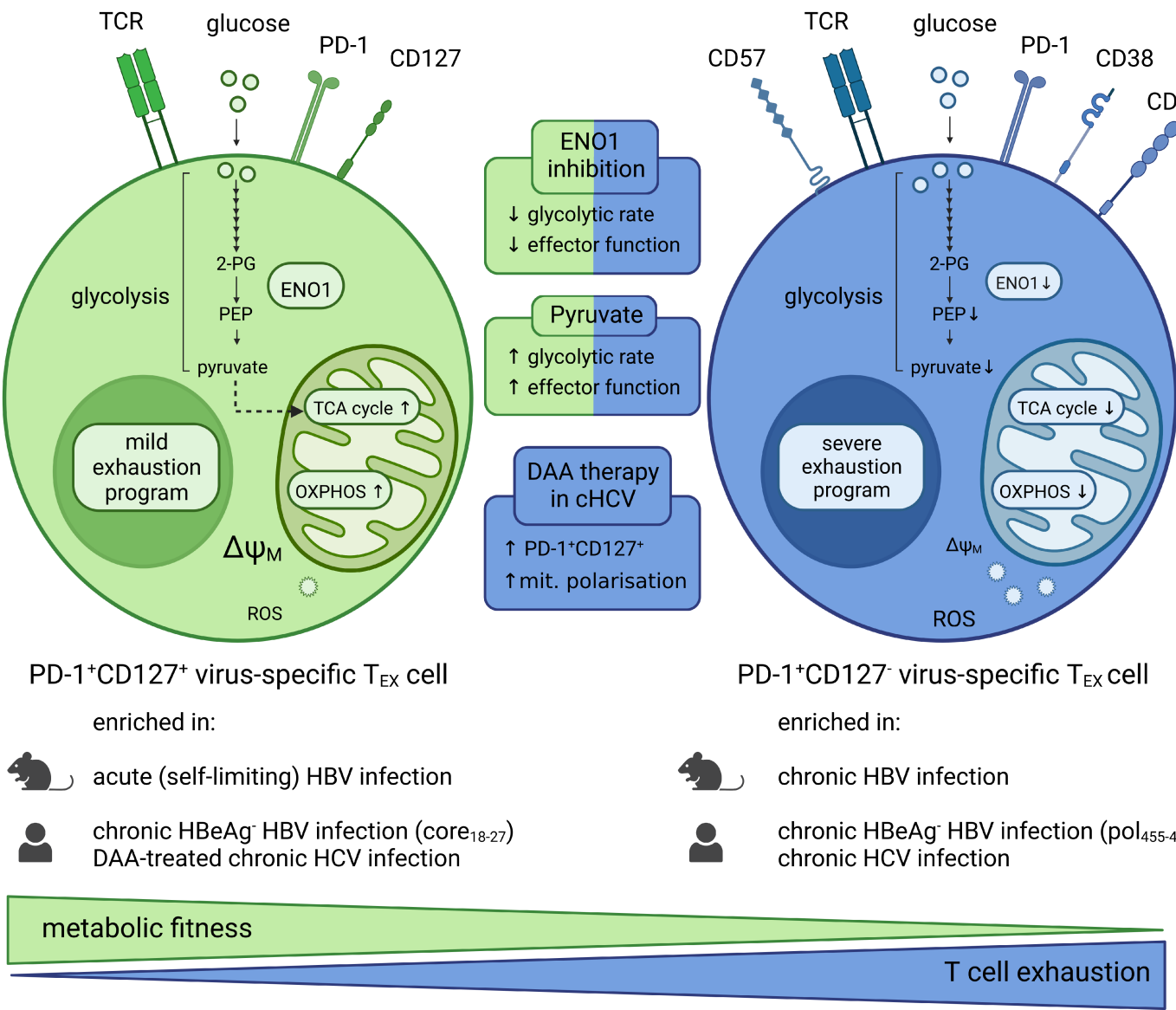

Supplement: Supplementary data [file gutjnl-2022-328734supp004.pdf]
